# The mitochondrial localized CISD-3.1/CISD-3.2 proteins are required to maintain normal germline structure and function in *Caenorhabditis elegans*

**DOI:** 10.1371/journal.pone.0245174

**Published:** 2021-02-05

**Authors:** Skylar D. King, Chipo F. Gray, Luhua Song, Ron Mittler, Pamela A. Padilla

**Affiliations:** 1 Division of Plant Sciences, College of Agriculture, Food and Natural Resources, Christopher S. Bond Life Sciences Center University of Missouri, Columbia, MO, United States of America; 2 Department of Surgery, University of Missouri School of Medicine, Christopher S. Bond Life Sciences Center University of Missouri, Columbia, MO, United States of America; 3 Department of Biological Sciences, University of North Texas, Denton, TX, United States of America; National Institute of Child Health and Human Development, UNITED STATES

## Abstract

Reproductive organs and developing tissues have high energy demands that require metabolic functions primarily supported by mitochondria function. The highly conserved CISD/NEET iron-sulfur (Fe-S) protein family regulates iron and reactive oxygen homeostasis, both of which are important for mitochondrial function. Disruption of iron and reactive oxygen homeostasis typically leads to detrimental effects. In humans, CISD dysfunction is associated with human health issues including Wolfram syndrome 2. Using *C*. *elegans*, we previously determined that the *cisd-1*, *cisd-3*.*1* and *cisd-3*.*2* have an overlapping role in the regulation of physiological germline apoptosis through the canonical programmed cell death pathway. Here, we isolated the *cisd-3*.*2(pnIs68)* mutant that resulted in physiological and fitness defects including germline abnormalities that are associated with abnormal stem cell niche and disrupted formation of bivalent chromosomes. The *cisd-3*.*2(pnIs68)* mutation led to complete disruption of the *cisd-3*.*2* gene expression and a decrease in expression of genetically intact *cisd-1* and *cisd-3*.*1* genes suggesting an indirect impact of the *cisd-3*.*2(pnIs68)* allele. The CISD-3.2 and CISD-3.1 proteins localize to the mitochondria in many tissues throughout development. The *cisd-3*.*2(pnIs68)* mutant displays phenotypes associated with mitochondrial dysfunction, including disruption of the mitochondrial network within the germline. These results further support the idea that the CISD protein family is required for mitochondrial function that supports important functions in animals including overall fitness and germline viability.

## Introduction

The highly conserved NEET proteins represented in humans as mitoNEET/CISD1, NAF-1/CISD2, and MiNT/Miner2/CISD3 constitute a novel class of iron-sulfur cluster proteins defined by their unique CDGSH amino acid sequence and their [2Fe-2S] cluster-binding domain [1–4]. The founding member of the NEET protein family, mitoNEET/CISD1, is localized to the outer mitochondrial membrane and binds to the anti-type 2 diabetes drug pioglitazone [2, 4]. Mammalian NAF-1/CISD2 localizes to the outer mitochondrial membrane, the endoplasmic reticulum (ER) and the membranes that connect the ER with mitochondria (*i*.*e*., mitochondrial associated membranes; MAM) [4]. The protein coded by CISD3/MiNT is localized to the inside of the mitochondrial matrix (MiNT for mitochondrial inner NEET Protein) [4]. The human MiNT protein has been crystalized and revealed to be a monomer with two distinct [2Fe-2S] cluster-binding domains [5].

Both MitoNEET/CISD1 and NAF1/CISD2 have a role in autophagy and apoptosis. The NAF1/CISD2 protein binds to Bcl-2 and this interaction is thought to regulate autophagy and apoptosis [6]. In addition to being involved in the regulation of autophagy and apoptosis, mitoNEET and NAF-1 were shown to regulate other cellular processes including reactive oxygen species (ROS) tolerance and signaling, calcium signaling, cell proliferation, electron transfer reactions, redox control and iron and iron-sulfur homeostasis [7]. NAF-1/CISD2 and/or mitoNEET/CISD1 dysfunction are associated with human disease conditions including cancer, diabetes, neurodegenerative diseases, obesity and heart disease [7]. In humans, CISD2 mutations result in the autosomal recessive disorder Wolfram syndrome 2 disease which is associated with juvenile diabetes mellitus, renal abnormalities, optic atrophy, neuropsychiatric disorders, and a greatly reduced life expectancy [8–11].

CISD1/mitoNEET and CISD2/NAF-1 have been extensively characterized in different cell systems and organisms, yet studies that focus on CISD3/MiNT in different biological systems are limited [1–5, 9]. Biological cell culture studies with CISD3/MiNT knockdown reveal an increase in mitochondrial labile iron accumulation and mitochondrial reactive species formation [5]. MiNT was also shown to transfer its [2Fe-2S] clusters to the human mitochondrial matrix ferrodoxins (FDX1 and FDX2) which are required for regulating iron and reactive oxygen species levels in the mitochondria [5]. These results suggest that CISD3/MiNT has a role in mitochondria homeostasis. Although the mitoNEET/CISD1, NAF-1/CISD2, and CISD3/MiNT proteins share sequence similarity and localization to the mitochondria, it is possible that the CISD proteins have distinct functions within the mitochondria [4, 5, 12].

Within *C*. *elegans* there are three genes that code for proteins that show homology to the human CISD proteins [12, 13]. The CISD-1 protein has homology to the mammalian NEET/CISD1 and NAF-1/CISD2 proteins. Our previous studies identified a role for the CISD protein family in physiological germline apoptosis through the canonical programmed cell death pathway in *C*. *elegans* [14, 15]. Specifically, disruption of *cisd-1* function resulted in an increase in cell corpses within the adult germline. The increased germline cell death is dependent on caspase/CED-3 and APAF-1/CED-4 function. Furthermore, the increased germline cell death observed in the *cisd* deficient animals was facilitated by the Bcl-2/CED-9 binding protein CED-13 [16]. This work places the CISD-1 protein as having a role in regulating physiological germline programmed cell death. Others have shown that *cisd-1* dysfunction in *C*. *elegans* results in hyperfused mitochondrial morphology, higher levels of generated mitochondrial superoxide, and a decrease level of ATP [17]. Additionally, *cisd-1* dysfunction negated the glucose-lowering response induced by the anti-diabetic drugs thiazolidinedione and pioglitazone in comparison to wild-type worms treated with the drug [17]. Together, the data suggest a role for *cisd-1* in maintaining basal glucose levels and mitochondrial bioenergetics [17]. The *C*. *elegans* CISD-3.1 and CISD-3.2 proteins show homology to human Miner2/CISD3 [12]. We previously determined that knock-down of *cisd-3*.*1* or *cisd-3*.*2* through RNAi led to animals with various germline defects including a decreased number of oocytes, distal tip cell (DTC) migration defects (Mig phenotype), and an increase in the number of cell corpses within the germline [16]. Combined, these studies support the idea that the CISD proteins in *C*. *elegans* have a role in germline function and metabolic processes.

The function of mitoNEET/CISD1 and NAF-1/CISD2 has been studied in various model systems, however little is understood about the function of MiNT/CISD3. Here we used CRISPR to produce a *cisd-3*.*2* deletion mutant (*cisd-3*.*2(pnIs68*)) and the CISD-3.1 and CISD-3.2 protein reporters. The *cisd-3*.*2(pnIs68*) results in complete disruption of the *cisd-3*.*2* gene and this dysfunction led to severe phenotypes including germline defects, reproductive dysfunction, reduced fitness, and disruption of mitochondrial network within the *C*. *elegans* germline. Due to the severe defects observed in the *pnIs68* allele relative to the previously described analysis of the *cisd-3*.*2(RNAi)* animal, we assessed the expression of the *cisd-1* and *cisd-3*.*1* genes. We observed through RT-PCR a decrease in *cisd-1* and *cisd-3*.*1* expression despite the loci for these genes being intact. This suggests an indirect impact on the of the *cisd-3*.*2(pnIs68)* allele on the *cisd* gene family. This work further supports the role the CISD family has in mitochondrial functions and proper germ cell differentiation in the germline of *C*. *elegans*.

## Results

### The *cisd-3*.*2(pnIs68)* mutant displays abnormal germline development and function

**Fig 1:**
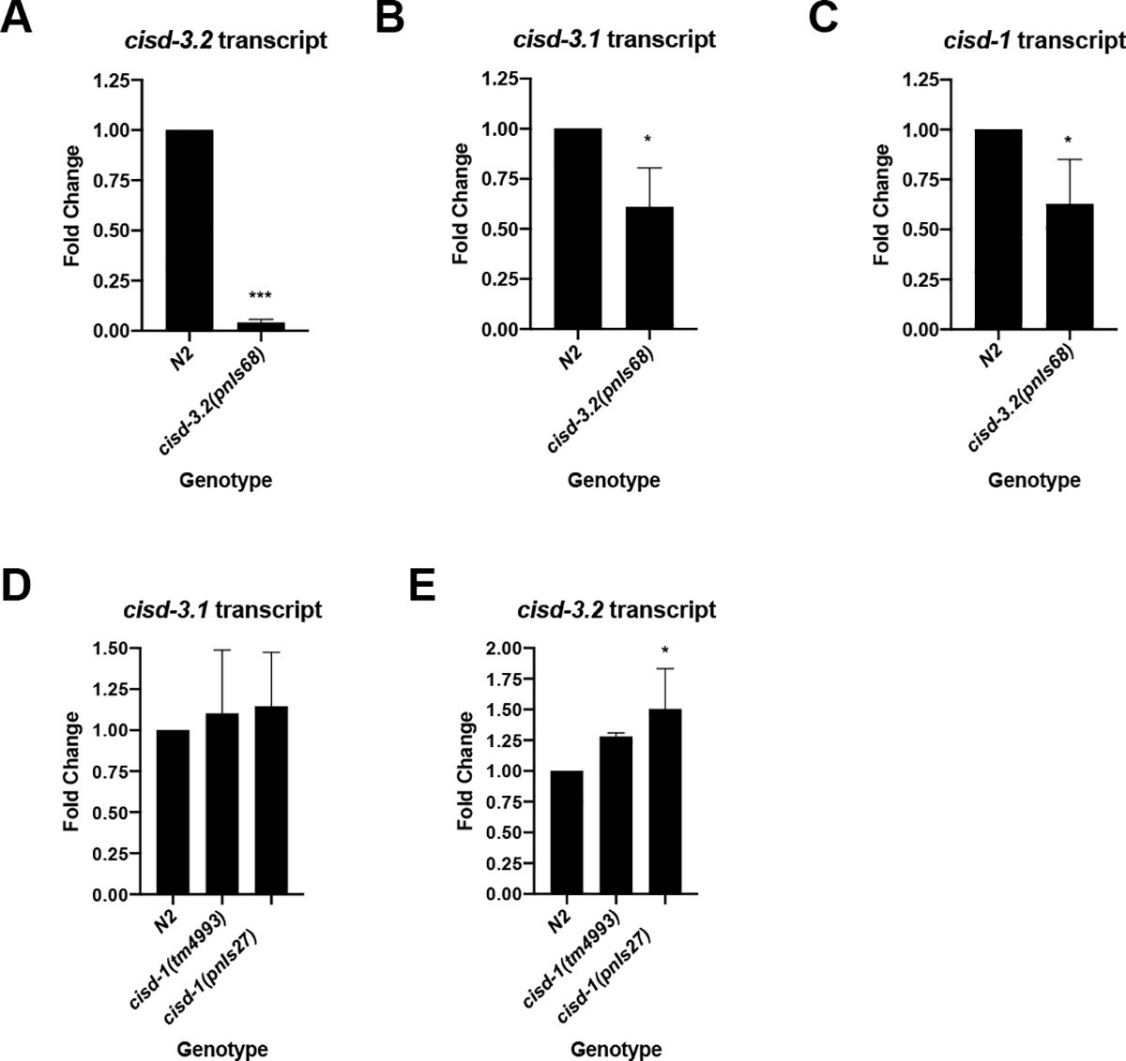
Deletion of *cisd-3*.*2* but not *cisd-1* reduces the expression of other *cisd* gene family members. **(A-C)** RT-PCR experiments to assess the fold change of the *cisd* transcripts in the *cisd-3*.*2(pnIs68)* mutant relative to N2 wild-type. **(A)** The *cisd-3*.*2* transcript is significantly reduced in the *cisd-3*.*2(pnIs68)* animal indicative of a null mutation. **(B)** The *cisd-3*.*1* transcript and **(C)** the *cisd-1* transcript is reduced but not absent in the *cisd-3*.*2(pnIs68)* animal**. (D-E)** RT-PCR experiments to assess the fold change of *cisd-3*.*1* and *cisd-3*.*1* in the *cisd-1(tm4993)* or *cisd-1(pnIs27)* mutant relative to N2 wild-type. **(A-E)** Data is from three independent experiments. Error bars represent standard deviations. The *** indicates P<0.0001; * indicates P<0.05 (unpaired two-tailed t test with Welch’s correction).

Previously we determined that deletion of the *cisd-1* gene and knock-down of *cisd-3*.*1* and *cisd-3*.*2* via RNAi resulted in various germline abnormalities including an increase in cell corpses within the adult germline and abnormal distal tip cell migration [16]. Given that RNAi results in a gene knock-down and such results in less severe phenotypes in comparison to genetic null mutants, we used CRISPR to produce a *cisd-3* mutant to better assess the phenotype(s) associated with CISD3 dysfunction. The *cisd-3*.*2(pnIs68)* mutant was isolated (S1 Fig) however, we were unable to obtain a stable *cisd-3*.*1* deletion mutant. RT-PCR analysis indicates that the *cisd-3*.*2(pnIs68)* allele is a *cisd-3*.*2* null mutation (Fig 1A). Molecular techniques (RT-PCR and genome sequencing) were used to assess if the *cisd-3*.*1* or *cisd-1* genes are impacted by the *cisd-3*.*2(pnIs68)* allele. DNA sequencing of the *cisd-3*.*1* and *cisd-1* loci in the *cisd-3*.*2(pnIs68)* mutant confirmed that there was not untargeted deletion of the *cisd-3*.*1* or *cisd-1* genes. However, we did observe a decrease in expression of *cisd-3*.*1* and *cisd-1* within the *cisd-3*.*2(pnIs68)* mutant (Fig 1B and 1C). We did not observe decreased expression of *cisd-3*.*1* or *cisd-3*.*2* in the *cisd-1* mutant (Fig 1D and 1E). In fact, there was a slight increase in *cisd-3*.*2* expression in the *cisd-1(pnIs27)* mutant. Combined, these data suggest that 1) *cisd-1 and cisd-3*.*1* genes do not compensate for deletion of *cisd*-3.2; 2) deletion of the *cisd-3*.*2* gene indirectly impacts the expression of *cisd-3*.*1* and *cisd-1* and 3) *cisd-3*.*2* may compensate for disruption of *cisd-1*.

**Fig 2:**
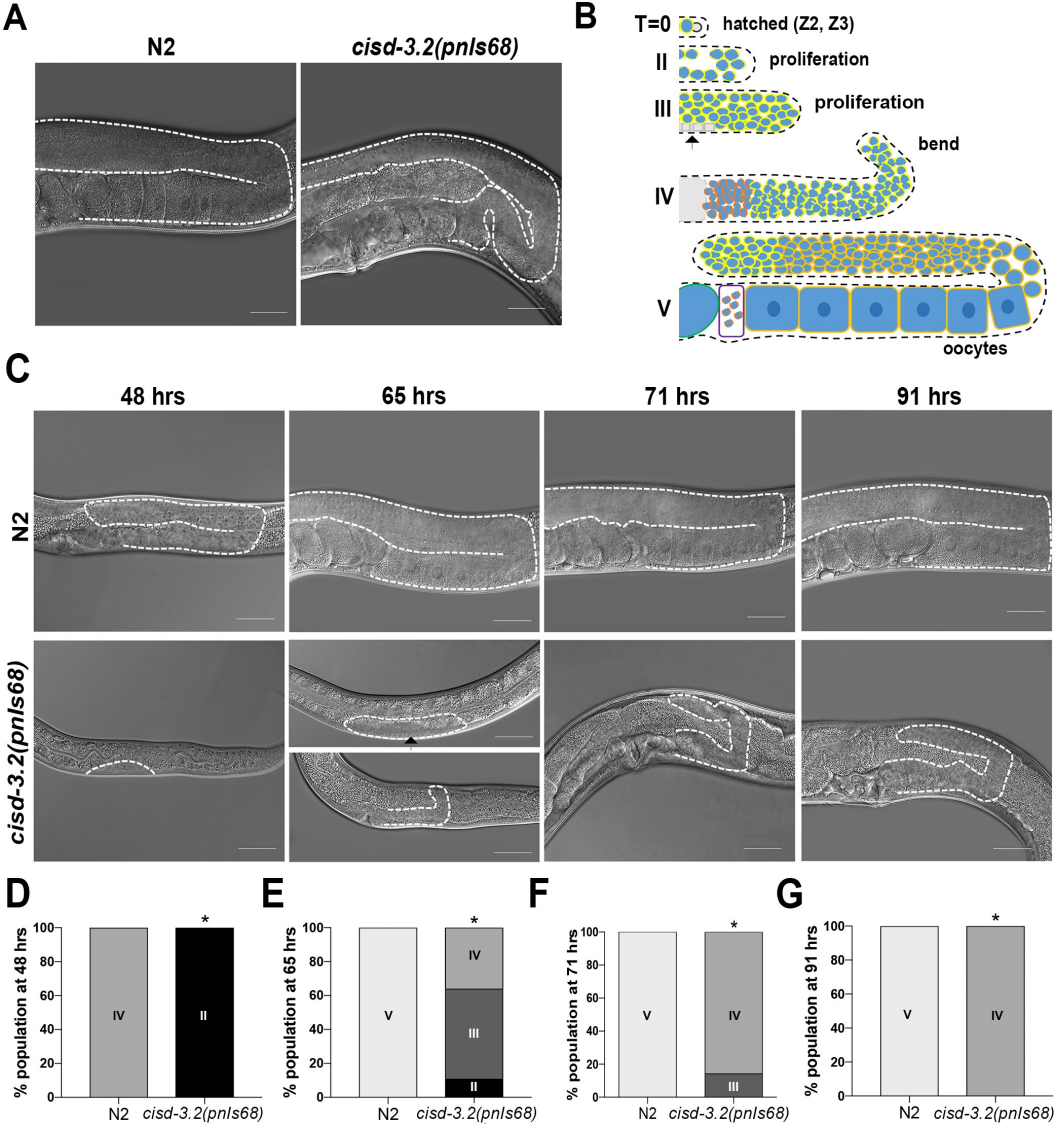
The *cisd-3*.*2(pnIs68)* allele leads to an abnormal germline structure and development phenotype. **(A)** Representative germline images of N2 wild-type and *cisd-3*.*2(pnIs68)* gravid adult hermaphrodite. For each animal, the gonad is outlined with a dashed white line. Scale bar indicates 20 μm. **(B)** Schematic diagram of a single gonad arm to note how the germline was categorized to assess gonad developmental progression. Time zero is at hatching when the germline contains Z2 and Z3 cells. At specific times post hatching, germlines were examined and categorized for initial germ cell proliferation (Class II), moderate germ cell proliferation and the presence of precursor vulva cells (black arrow) (Class III), the formation of a bend within the gonad (Class IV), or the presence of oocytes (Class V). **(C)** Representative image of the germline within the N2 wild-type or *cisd-3*.*2(pnIs68)* hermaphrodite at specified time points. The gonad is outlined with a dashed white line. Black arrow indicates the presence of precursor vulva cells. **(D-G)** The germline of N2 wild-type and *cisd-3*.*2(pnIs68)* hermaphrodite, at specified time points, was examined and classified. At each time point, the percentage of the population for the specified stage of development was determined. Shown is the mean from three independent experiments (n = 30). The * indicates significant difference; P value <0.0001, (Fisher’s exact two-sided test).

Phenotypic analysis was conducted to gain a deeper understanding of the impact of the *cisd-3*.*2(pnIs68)* allele. The *cisd-3*.*2(pnIs68)* adult hermaphrodite exhibits an abnormal adult germline morphology (Fig 2A). In N2 wild-type hermaphrodites, the germline progresses through developmental stages that ultimately produces mature sperm and oocytes [18]. The cartoon diagram in Fig 2B communicates the progression of N2 wild-type germline development based on the extent of germ cell proliferation, bend of the germline, and presence of sperm and oocytes. At specific time points (48, 65, 71, and 91 hours post hatching) we visualized and classified the germline development for the *cisd-3*.*2(pnIs68)* and N2 wild-type hermaphrodite (Fig 2C–2G). The *cisd-3*.*2(pnIs68)* animal exhibits a significant delay in germline development relative to the N2 wild-type hermaphrodite (Fig 2C). At 48 hours post hatch, the N2 wild-type germline displays a bend in the gonad, which is indicative of the L4 stage of development. Whereas, the germline of the *cisd-3*.*2(pnIs68)* animal has minor germ cell proliferation (Fig 2C and 2D). By 65 hours post hatch, the N2 wild-type animal contains mature oocytes and embryos within the uterus (Class V). Whereas, the germline of the *cisd-3*.*2(pnIs68)* animal is at various stages and the germline contains some germ cell proliferation (Class III) and some of the animals display a small germline with a bend (Class IV) (Fig 2C and 2E). At 71 and 92-hours post hatching, the germline within the *cisd-3*.*2(pnIs68)* animal continues to show a developmentally delayed, abnormal germline that visibly lacks oocytes (Fig 2C, 2F and 2G). These data suggest that complete disruption of *cisd-3*.*2* and consequential decreased expression of *cisd-3*.*1* and *cisd-1* leads to severe germline defects.

**Fig 3:**
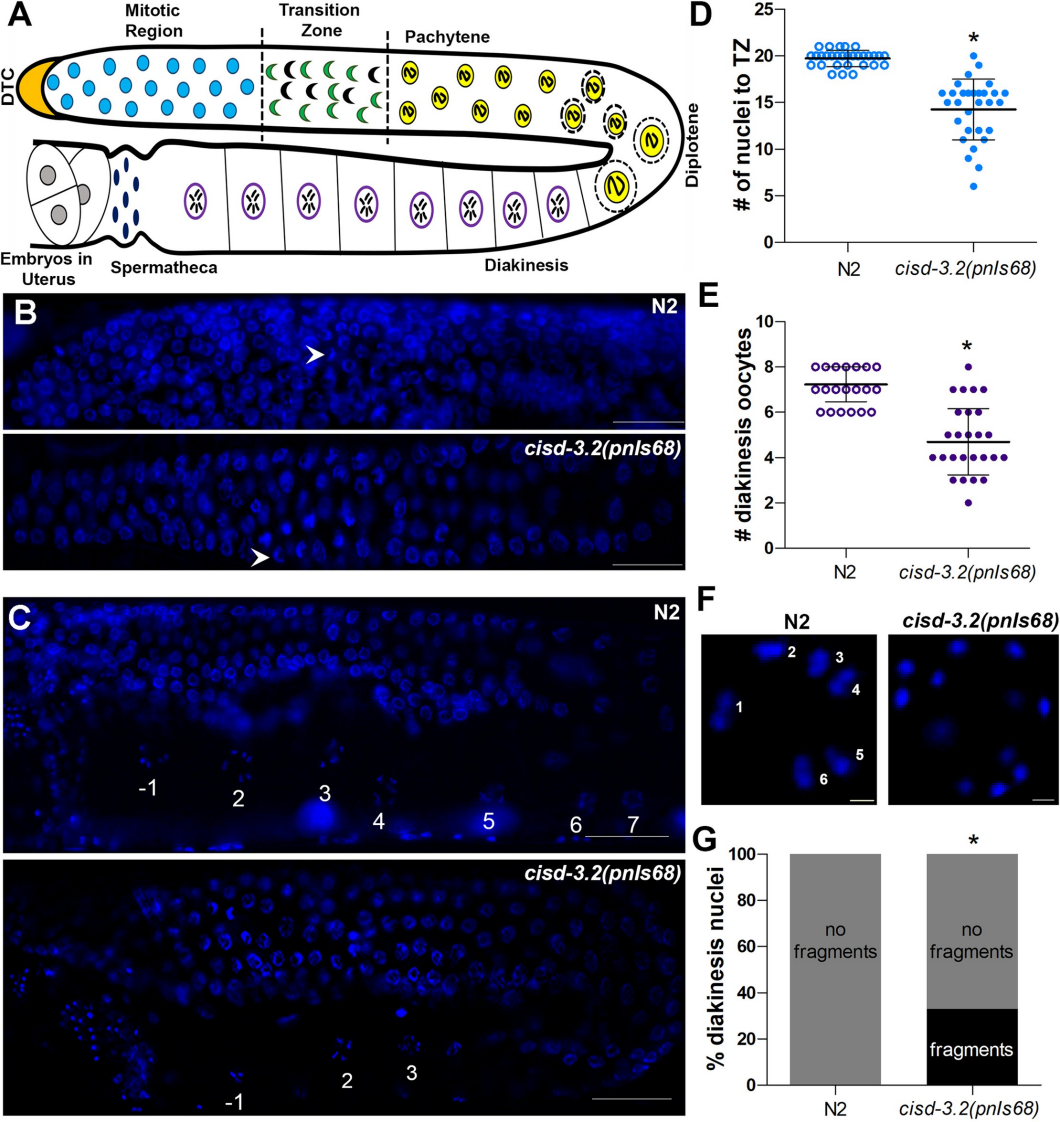
The *cisd-3*.*2(pnIs68*) mutant displays multiple germline defects. **(A)** Schematic diagram of the meiotic events within the adult N2 wild-type hermaphrodite germline. The mitotic cells proximal to the distal tip cell (DTC) progress entering meiosis to produce oocytes and sperm which are contained within the spermatheca. **(B)** Representative image of the mitotic region and transition zone, within the N2 wild-type or *cisd-3*.*2(pnIs68)* 1-day old adult hermaphrodite germline. The white arrowhead points to the distinct crescent shaped nuclei that notes the switch from mitosis to meiosis. Scale bar represents 10 μm. **(C)** Representative images of the pachytene, diplotene and diakinesis region, within the germline of N2 wild-type or *cisd-3*.*2(pnIs68)* 1-day old adult hermaphrodite animal. The number of oocytes within the diakinesis region are numbered; -1 indicates the primary oocyte. Scale bar represents 10 μm. **(D)** Quantification of the number of nuclei from the DTC region to the transition zone (TZ) in the N2 wild-type or *cisd-3*.*2(pnIs68)* 1-day old adult hermaphrodite germline (n = 30); * indicates P value ≤ 0.0001, (unpaired two-tailed t test with Welch’s correction). **(E)** Quantification of the number of oocytes in the diakinesis region of the N2 wild-type or *cisd-3*.*2(pnIs68)* hermaphrodite animal (n = 30); * indicates P value ≤ 0.0001, (unpaired two-tailed t test with Welch’s correction). **(F)** Representative image of oocyte DNA nuclei within the N2 wild-type or *cisd-3*.*2(pnIs68)* 1-day old adult hermaphrodite germline. The number of bivalent chromosomes is noted for N2 wild-type animals. This particular oocyte nuclei from the *cisd-3*.*2(pnIs68)* animal contains twelve chromosomal fragments. Scale bar represents 1 μm. **(G)** The percentage of oocytes with nuclei containing no chromosomal fragments (six bivalent chromosomes, grey bar) or nuclei containing fragmented chromosomes (> six chromosomes, black bar) was determined in the N2 wild-type (n = 145) and *cisd-3*.*2(pnIs68)* (n = 111) 1-day old adult hermaphrodite germline; * indicates P value < 0.0001, (Fisher’s exact two-sided test). For all experiments, the chromosomal DNA was detected by staining with Hoescht 33342.

**Fig 4:**
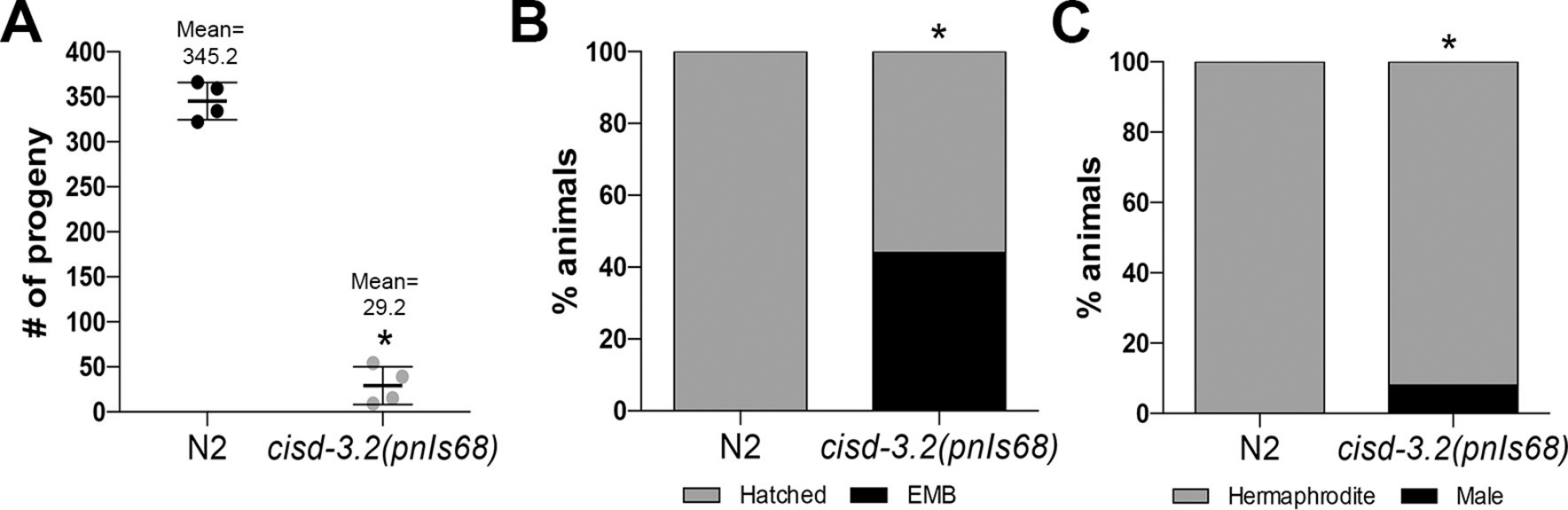
The *cisd-3*.*2(pnIs68)* mutant has a reduced fitness. **(A)** The *cisd-3*.*2(pnIs68)* animal has a significantly reduced number of progeny relative to N2 wild-type (n = 4); * indicates P<0.0001, (two-tailed student t-test with Welch’s Correction). error bars represent SD. **(B)** The *cisd-3*.*2(pnIs68)* animal has a significant increase in embryo lethality (EMB) relative to N2 wild-type (n = 9); * indicates P<0.0001, (Fisher’s exact two-sided test). **(C)** The *cisd-3*.*2(pnIs68)* animal has a significant increase in male progeny (High Incidence of Male phenotype, HIM) relative to N2 wild-type (n = 9); * indicates P<0.0001, (Fisher’s exact two-sided test).

To further evaluate the germline defect in the *cisd-3*.*2(pnIs68)* mutant we used Hoescht 33342 to examine the germline nuclei. This approach allows one to assess meiotic progression, in particular the stage of meiotic prophase, based on nuclei morphology within the hermaphrodite germline (Fig 3A) [19, 20]. The nuclei located in the mitotic region can be distinguished from the crescent-shaped nuclei within the transition zone (Fig 3A). Also, the length of the mitotic region can be determined by counting the number of nuclei diameters between the distal cell tip and the transition zone; the mitotic region length is approximately 20 nuclei diameters [20]. The mitotic region within the *cisd-3*.*2(pnIs68)* mutant appears to have less cells (Fig 3B). We quantified the length of the mitotic region and determined that it is variable and significantly reduced in the *cisd-3*.*2(pnIs68)* germline by 27.7% in comparison to the N2 wild-type germline (Fig 3D). Furthermore, within the c*isd-3*.*2(pnIs68)* germline the number of oocytes within the diakinesis region is significantly reduced by 35.0% relative to N2 wild-type animals (Fig 3C and 3E); note that a reduced number of oocytes within the diakinesis region was observed in the *cisd-3*.*2(RNAi)*, *cisd-3*.*1(RNAi) and cisd-1(tm4993)* animal [16]. Further inspection of the oocyte found that a significant proportion of the *cisd-3*.*2(pnIs68)* oocytes contain a number of chromosome fragments or univalent chromosomes instead of the typical six bivalent chromosomes (Fig 3F and 3G). This differs from the six bivalent chromosomes observed within the oocytes of the N2 wild-type adult hermaphrodite (Fig 3F and 3G). Although the *cisd-3*.*2(pnIs68)* population can be maintained, there is a significant decrease (91.5%) in fecundity (Fig 4A), a 44% increase in embryo lethality (Fig 4B), and an 8% increase in incidence of males (HIM phenotype) (Fig 4C) relative to the N2 wild-type control. Combined, these results further support the idea that the CISD family is essential for germline function and in particular germ cell development.

### The CISD-3 proteins are expressed in various tissues including the germline

**Fig 5:**
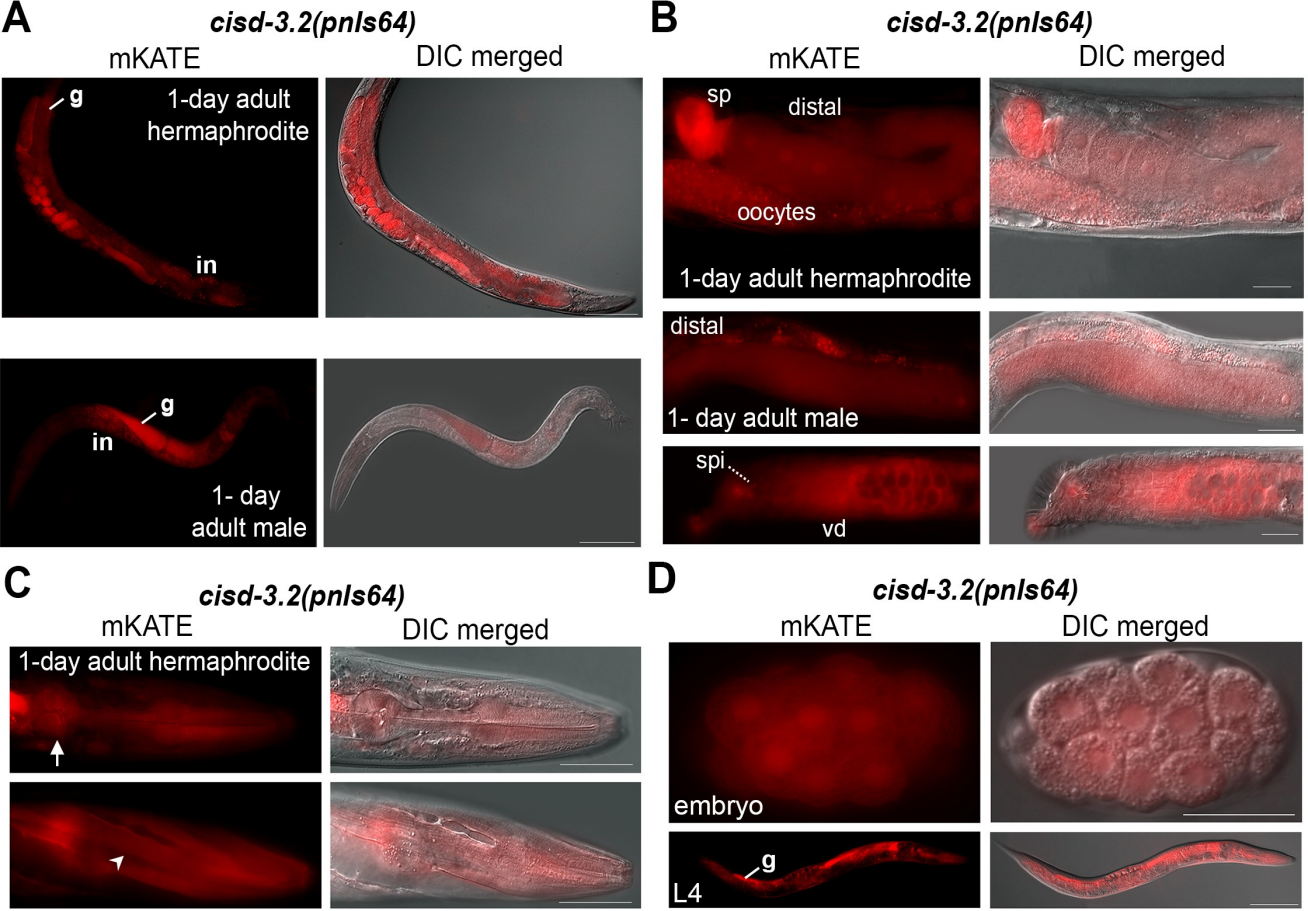
The *cisd-3*.*2p*::*mKATE* reporter is expressed in multiple tissues including the adult and larvae germline. Images shown include fluorescent and fluorescent merged with DIC. **(A-B)** Representative images of the *cisd-3*.*2(pnIs64)* adult hermaphrodite or male as indicated. Organs and germline regions within the hermaphrodite is noted as germline (g), expression throughout the intestine is noted as (in), oocytes, distal germline region, and spermatheca (sp). The distal region of the gonad (distal), vas deferens (vd) and spicule (spi) is indicated in the adult male. Scale bar indicates 50 μm. **(C)** Representative image of the anterior region of the *cisd-3*.*2(pnIs64)* adult hermaphrodite. mKATE expression is observed in the pharynx (white arrow) and body wall muscle (white arrow head). Scale bar indicates 50 μm. **(D)** Representative images of the *cisd-3*.*2(pnIs64)* embryo and L4 larval stage. mKATE expression is observed throughout the embryonic blastomeres, and within the intestine, pharynx region and developing germline of larvae. Scale bar indicates 100 μm.

To examine *cisd-3*.*2* expression we used CRISPR to produce a *cisd-3*.*2* promotor (*cisd-3*.*2p*) transcriptional reporter (*cisd-3*.*2(pnIs64)*) (S1 Fig). The *cisd-3*.*2p*::*mKATE* expression is present in the germline of the adult hermaphrodite and male (Fig 5A). Within the hermaphrodite, *cisd-3*.*2p*::*mKATE* is expressed throughout the germline including the distal gonad region, oocytes, and spermatheca (Fig 5B). Within the adult male *cisd-3*.*2p*::*mKATE* expression is observed in the distal gonad, spicule and vas deferens (Fig 5B). Note that the mKATE reporter disrupts *cisd-3*.*2* function (S1 Fig) and thus the male germline looks abnormal (Fig 5B). The *cisd-3*.*2p*::*mKATE* expression occurs within other tissues including the pharynx and the anterior body wall muscle of the adult hermaphrodite (Fig 5C). The *cisd-3*.*2p*::*mKATE* expression was also observed in the embryo blastomeres and within the developing germline and intestine of larvae (Fig 5D). These expression profiles support the idea that *cisd-3*.*2* has a functional role in both the male and hermaphrodite and within multiple tissues including the germline.

**Fig 6:**
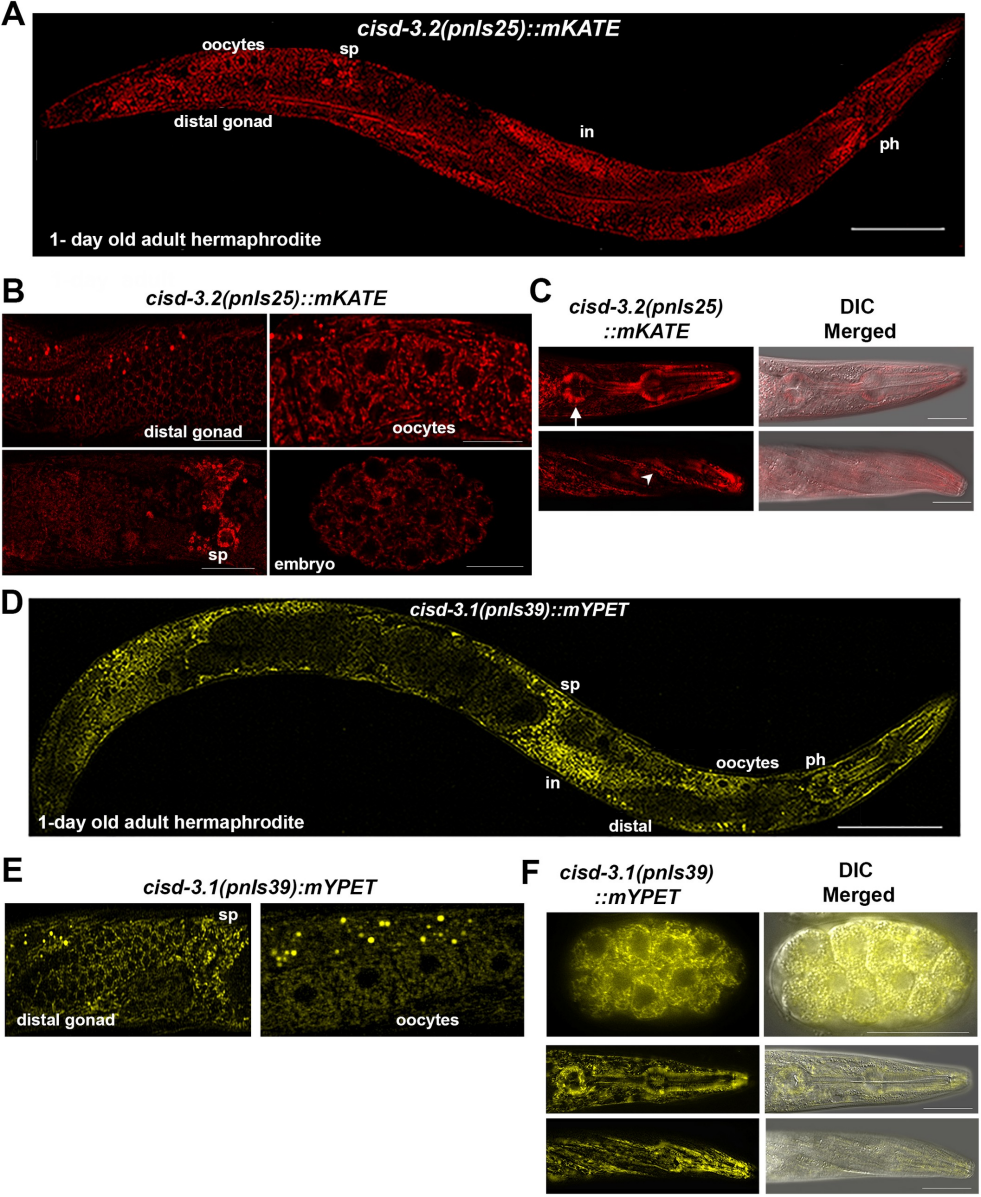
The CISD-3.2::mKATE and CISD-3.1::mYPET reporters are expressed in multiple tissues. The *cisd-3*.*2p*::*CISD-3*.*2*::*mKATE* and *cisd-3*.*1p*::*CISD-3*.*1*::*mYPET* strains were produced via CRISPR. **(A)** The *cisd-3*.*2p*::*CISD-3*.*2*::*mKATE* fluorescent translational reporter is expressed in many adult hermaphrodite tissues including the intestine, oocytes, distal gonad region, and spermatheca (sp) in the one day old adult hermaphrodite. Scale bar indicates 100 μm. **(B)**. Representative images of *cisd-3*.*2p*::*CISD-3*.*2*::*mKATE* reporter expression in multiple tissues including portions of the germline, such as the distal gonad, oocytes and spermatheca (sp), and within the embryo. Scale bar indicates 50 μm (images of germline region) or 10 μm (image of embryo embryo). **(C)** Representative image of the anterior region of the 1-day old adult hermaphrodite. Images shown include fluorescent and fluorescent merged with DIC. The *cisd-3*.*2p*::*CISD-3*.*2*::*mKATE* expression is observed in the pharynx (white arrow) and body wall muscle (white arrow head). Scale bar indicates 50 μm. **(D)** The *cisd-3*.*1p*::*CISD-3*.*1*::*mYPET* reporter is expressed in many adult hermaphrodite tissues including the pharynx (ph), intestine (in), oocytes, distal gonad region, and spermathecal (sp) in the 1-day old adult hermaphrodite. Scale bar represents 100 μm. **(E)** Representative images of *cisd-3*.*1p*::*CISD-3*.*1*::*mYPET* fluorescent translational reporter expression in the distal gonad, spermatheca (sp) and oocytes. Scale bar indicates 50 μm. **(F)** Representative images of *cisd-3*.*1p*::*CISD-3*.*1*::*mYPET* expression in the embryo, pharynx region, or muscle region. Images shown include fluorescent and fluorescent merged with DIC. Scale bar indicates 10 μm (image of embryo) or 50 μm (images of anterior region).

To assess the localization of CISD-3.2 and CISD-3.1, we used CRISPR to produce the *cisd-3*.*2p*::*cisd-3*.*2*::*mKATE* and *cisd-3*.*1p*::*cisd-3*.*1*::*mYPET* translational reporter strains (S2A and S2B Fig) [26]. The CISD-3.2::mKATE reporter is detected in many tissues of the adult hermaphrodite (Fig 6A). Specifically, the CISD-3.2::mKATE expression is observed in the distal gonad, oocytes, spermatheca, and sperm within the hermaphrodite germline as well as throughout the embryo blastomeres (Fig 6B). Additionally, CISD-3.2::mKATE expression is observed in the pharynx and body wall muscle within the head region of the adult hermaphrodite (Fig 6C). Similar to the expression observed for the CISD-3.2::mKATE reporter, the CISD-3.1::mYPET reporter is detected in various tissues throughout the adult hermaphrodite (Fig 6D). Specifically, the CISD-3.1::mYPET reporter is detected in the germline including the distal gonad region, spermatheca, sperm and oocytes (Fig 6E). Furthermore, the CISD-3.1::mYPET expression is observed throughout the embryo blastomeres and throughout the adult pharynx and body wall muscle in the head region (Fig 6F).

**Fig 7:**
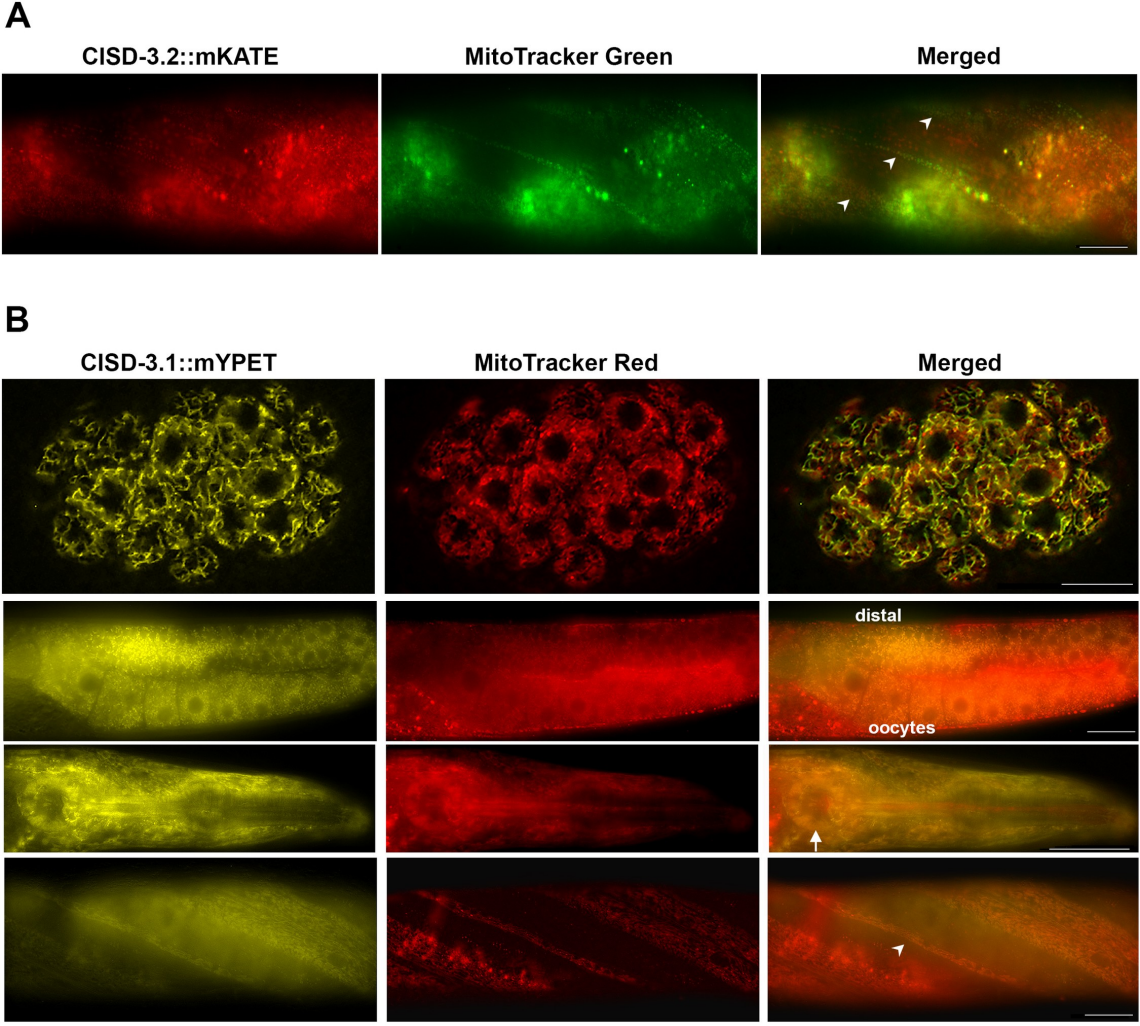
The CISD-3.2::mKATE and CISD-3.1::mYPET reporters colocalize with mitochondria in multiple tissues. **(A)** Representative image of the body wall muscle region in an adult *cisd-3*.*2(pnIs25)*::*mKATE* hermaphrodite showing colocalization of CISD-3.2::mKATE and MitoTracker Green FM (white arrowhead). Scale bar represents 50 μm. **(B)** Representative images of *cisd-3*.*1(pnIs39)*::*mYPET* animals showing colocalization of CISD-3.1::mYPET and MitoTracker Red CMXRos within the embryo (first panel), adult germline (second panel), pharynx region (third panel), and body wall muscle (fourth panel). Scale bar in image with embryo represents 10 μm. Scale bar in image with the adult animal represents 50 μm.

The CISD-3.2::mKATE and CISD-3.1::mYPET puncta resemble mitochondria (Fig 6). Therefore, we conducted colocalization analysis with MitoTracker Green FM or MitoTracker Red. We determined that the CISD-3.2::mKATE expression colocalizes with MitoTracker Green FM throughout the body wall muscle in the adult hermaphrodite (Fig 7A, white arrowhead). Additionally, the CISD-3.1::mYPET expression colocalizes with MitoTracker Red in various tissues including the embryonic blastomeres (Fig 7B, first panel), adult hermaphrodite germline (Fig 7B, second panel), anterior head region including the pharynx (Fig 7B, third panel, white arrow), and body wall muscle (Fig 7B, fourth panel, white arrowhead). These results indicate that the CISD-3.2 and CISD-3.1 proteins localize to the mitochondria of various tissues.

### The *cisd-3*.*2(pnIs68)* animal displays phenotypes associated with mitochondrial dysfunction

**Fig 8:**
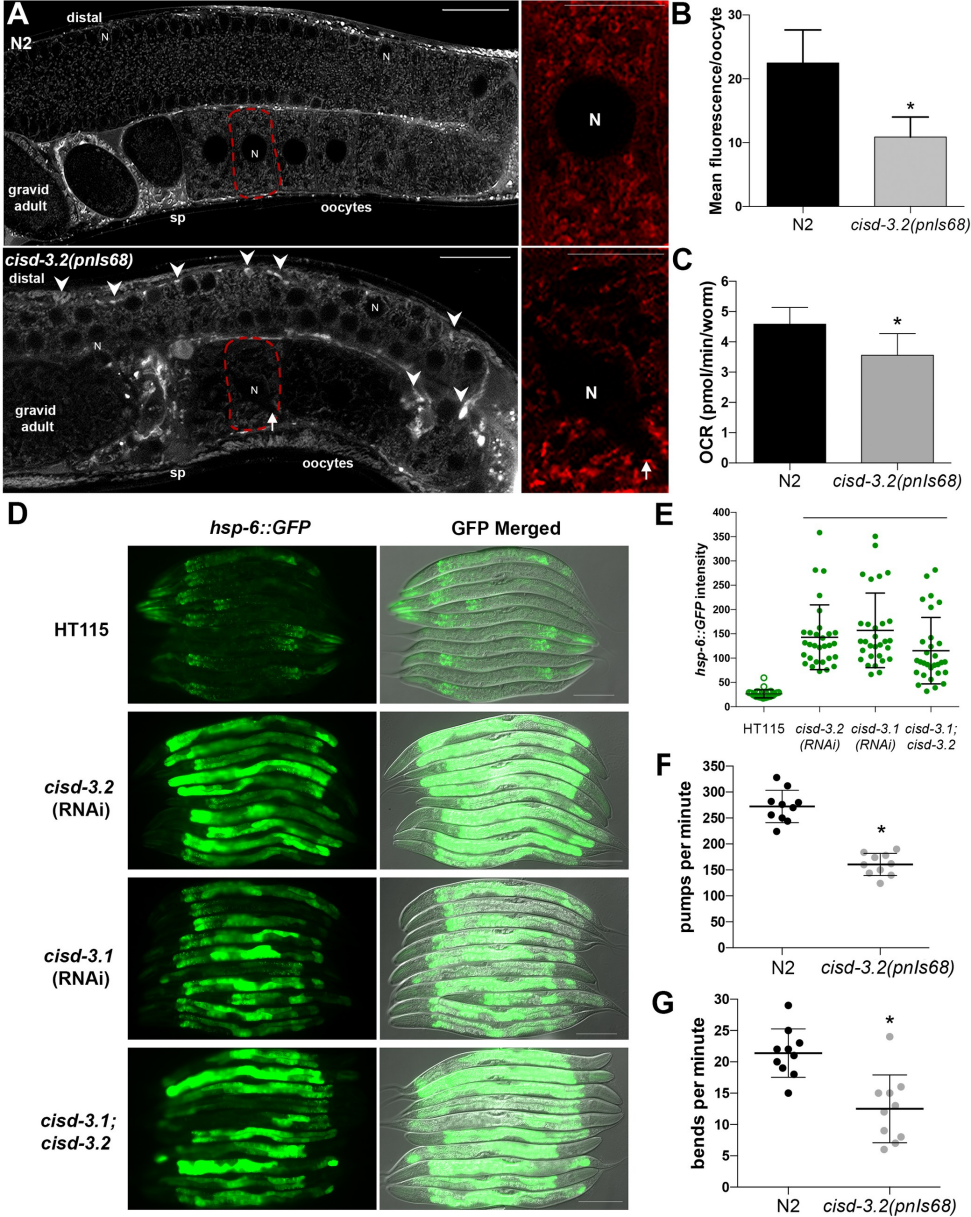
CISD dysfunction results in abnormal mitochondrial function. **(A)** The mitochondria, detected using MitoTracker Red CMXRos, within the germline of the young gravid *cisd-3*.*2(pnIs68)* animal has abnormal structure and distribution in comparison to the developmentally matched N2 wild-type germline. Shown is the distal gonad, oocytes, and spermatheca (sp) region of the gonad; a single oocyte is indicated by dashed lined and enlarged in the right panel. The mitochondria are within the cytoplasm and surround the nuclei region (N). Arrowhead shows regions where “globular” structures accumulate. Arrow points to oocyte region void of MitoTracker Red CMXRos staining in the *cisd-3*.*2(pnIs68)* mutant germline. Scale bar indicates 50 μm (whole germline image) or 25 μm (oocyte image). Analysis of 15 animals from three independent experiments was conducted. **(B)** The *cisd-3*.*2(pnIs68)* animal (gravid adult) has a decrease in mean fluorescence intensity per oocyte relative N2 wild-type. The fluorescence, using MitoTracker Red CMXRos, was quantified in single oocytes of the *cisd-3*.*2(pnIs68)* mutant and the N2 wild-type animal; * indicates P value<0.0001, (unpaired t-test with Welch’s correction). Oocytes from three independent experiments for a total of 15 oocytes per genotype were analyzed. The error bar represents standard deviation. **(C)** The *cisd-3*.*2(pnIs68)* mutant has a decrease in oxygen consumption rate relative to N2 wild-type animals; * indicates P<0.003, (unpaired t-test with Welch’s correction). Animals assayed were non-gravid young adults. **(D)** Representative group images of the *hsp-6p*::*gfp* reporter strain fed specified RNAi food. HT115 is the control RNAi strain (empty vector). The images shown include fluorescent and fluorescent merged with DIC. Animals assayed were L4 larvae. Scale bar indicates 100 μm. **(E)** Quantification of *hsp-6*::*GFP* intensity in *cisd-3*.*2(RNAi)* and/or *cisd-3*.*1(RNAi)* relative to control animals fed HT115 control RNAi strain (n = 30). Bar indicates P<0.0001 (ordinary one-way ANOVA, Tukey’s multiple comparisons test). Error bars represent standard deviation. **(F)** The pharyngeal pumping rate per minute is decreased in the *cisd-3*.*2(pnIs68)* animal relative to N2 wild-type (n = 10); * indicates P<0.0001, (two-tailed student t-test with Welch’s Correction). Animals assayed were non-gravid young adults. **(G)** The rate of locomotion, determined by the number of body bends per minute, is reduced in the *cisd-3*.*2(pnIs68)* animal relative to N2 wild-type (n = 10); * indicates P = 0.0006, (two-tailed student t-test with Welch’s Correction). Animals assayed were non-gravid young adults.

The phenotype and expression analyses shown thus far support the idea that the CISD-3 proteins functions within the mitochondria of many tissues including the germline. To assess if *cisd-3*.*2(pnIs68)* impacts mitochondrial structure within the germline, we stained the *cisd-3*.*2(pnIs68)* mutant hermaphrodite with MitoTracker Red CMXRos, which is a membrane-potential-dependent mitochondrial dye. The mitochondria within the N2 wild-type animal, has a consistent pattern within the syncytium and a mesh-like structure within the oocytes (Fig 8A). The localization pattern is consistent with previous MitoTracker Red CMXRoS staining experiments conducted in *C*. *elegans* [21–23]. Within the germline of the *cisd-3*.*2(pnIs68)* hermaphrodite, the MitoTracker Red CMXRoS staining pattern is abnormal with an aggregated distribution (Fig 8A, white arrowhead). Furthermore, within the *cisd-3*.*2(pnIs68)* oocyte, the MitoTracker Red CMXRos staining was not uniform, for example, regions were void of staining (Fig 8A, white arrow) and there is a significant decrease in fluorescent level within the oocytes by 51.6% (Fig 8B).

The abnormal staining pattern of MitoTracker Red CMXRos, as a membrane-potential-dependent mitochondrial dye, within the *cisd-3*.*2(pnIs68)* animal suggests that there is a decrease in mitochondrial membrane potential. We therefore examined if the *cisd-3*.*2(pnIs68)* allele impacts mitochondrial function. Relative to the N2 wild-type adult hermaphrodite, the *cisd-3*.*2(pnIs68)* hermaphrodite has a decrease rate of basal oxygen consumption (Fig 8C). The *hsp-6p*::*GFP* reporter was used, which has previously been used to demonstrate the mitochondrial unfolding response, to assess if the mitochondrial unfolding protein response (UPRmt) is induced in animals (L4 stage) with decreased *cisd* function, relative to control [24]. We opted to use RNAi so we could assess knockdown of *cisd-3*.*1* and *cisd-3*.*2*. Relative to control, the expression of the *hsp-6p*::*GFP* reporter is increased in the *cisd-3*.*2(RNAi)* and *cisd-3*.*1(RNAi)* animal relative to control (Fig 8D and 8E). The combination of *cisd-3*.*2* and *cisd-3*.*1* knockdown did not further increase the expression of the *hsp-6p*::*GFP* reporter relative to knockdown of a single *cisd-3* gene (Fig 8E). Additional phenotypes were observed in *cisd-3*.*2(pnIs68)* mutants including a reduced lifespan (S3 Fig). Furthermore, there were defects in pharyngeal pumping (Fig 8F) and motility (Fig 8G) in the *cisd-3*.*2(pnIs68)* mutant. Taken together, these results indicate that CISD function is essential for mitochondrial function. Furthermore, the *cisd-3*.*1(pnIs68)* allele, which results in complete disruption of *cisd-3*.*2* and partial decrease in *cisd-3*.*1* and *cisd-1* expression, impact functions associated with energy-requiring processes.

## Discussion

The CISD/NEET proteins are highly conserved and present within the three domains of life [12, 13]. Previous biochemical studies have shown that MiNT/CISD3 can transfer it’s [2Fe-2S] clusters to the mitochondrial matrix proteins FDX1 and FDX2 [5]. Mammalian FDX1 is involved with adrenal steroidogenesis, bile acid, vitamin D synthesis and the reduction of mitochondrial CYP 450 proteins (CYP11A1, CYP11B) [25, 26]. FDX2 can accept electrons from NADPH, contains a [2Fe-2S] cluster but does not reduce cytochrome P450 [25]. Similar to knockdown of CISD3/MiNT in cells, knockdown of FDX1 and FDX2 results in iron accumulation within the mitochondria [25, 27]. Thus, it is likely that the disruption of CISD3 function in a whole organism can have detrimental impacts due to altered iron homeostasis and mitochondrial dysfunction.

Here we show that the *C*. *elegans* CISD-3 proteins (CISD-3.2 and CISD-3.1) are localized to the mitochondria in many tissues, throughout development, and in both males and hermaphrodites. Knockdown of *cisd-3*.*2* and *cisd-3*.*1* using RNAi results in an increase in germline apoptosis [16], and an increase in the *hsp-6*:*GFP* reporter for mitochondrial unfolded protein response (mtUPR) (Fig 8D and 8E). The *cisd-3*.*2(pnIs68)* mutant has a more severe phenotype relative to the *cisd-3*.*2(RNAi)* animal. This could be due to the complete reduction in *cisd-3*.*2* transcript and an indirect impact on the expression of both *cisd-3*.*1* and *cisd-1* genes (Fig 1). Additional studies are needed to determine why the transcript levels of *cisd-3*.*1* and *cisd-1* are reduced in the *cisd-3*.*2(pnIs68)* animal. One intriguing idea to consider is that the absence of *cisd-3*.*2* induces mitochondrial dysfunction (e.g. mtUPR) which in turn impacts mitochondria-to-nuclear communication. A decrease in *cisd-3*.*1* or *cisd-3*.*2* was not observed in the *cisd-1* mutants (Fig 1) therefore, the *cisd* genes may not have overlapping function. It remains to be seen if reduction of *cisd-3*.*1* impacts the expression of the other *cisd* genes. We hypothesize that disruption of all three *cisd* genes leads to severe mitochondrial defects (perhaps lethality). The *cisd-3*.*2(pnIs68)* mutant has a severely dysfunctional germline and a significantly reduced viability and lifespan. Furthermore, of the few *cisd-3*.*2(pnIs68)* animals that do survive and reach adulthood, the mitochondria within their germline are abnormally distributed (Fig 8). Within *C*. *elegans*, mitochondrial dysfunction will impact the electron transport chain, upregulate the mtUPR response, decrease oxygen consumption rate, and at times alter lifespan [28–31]. We hypothesize that *cisd* dysfunction disrupts mitochondria homeostasis which in turn impacts iron homeostasis and metabolic processes, thus, negatively impacting energy requiring processes including development and meiosis. It will be of interest to examine the specific proteins that the *C*. *elegans* CISD proteins interact with and if the function of these proteins is vital for normal germline function.

The *C*. *elegans* hermaphrodite germline rapidly produces a population of sperm, followed by oocytes over a period of several days [18, 19, 32]. The distal end of the adult germline provides a stem cell niche necessary for mitotic cell proliferation (Fig 3A). The germ cells located in the mitotic region replenish themselves and generate cells that leave the niche and enter into meiosis and gametogenesis; thus they fit the criteria of a stem cell [19]. The *cisd-3*.*2(pnIs68)* mutant has a variable and significantly reduced number of nuclei from the DTC region to the transition zone (Fig 3B and 3D). This suggests that the *cisd-3*.*2(pnIs68)* mutant has abnormalities within the stem cell niche region of the gonad. It will be of interest to further examine the role of mitochondria homeostasis with the maintenance of the stem cell niche. The stages of meiotic prophase occur along the distal-proximal axis of the adult gonad (Fig 3A) [33]. Within N2 wild-type hermaphrodites, during diakinesis, the six individual bivalents are visualized in oocytes as the nuclear volume increases and chromosome condensation proceeds. Unattached univalent chromosomes can be observed when meiotic dysfunction occurs such as abnormal chiasmata [34]. The pairing of homologous chromosomes and the formation of bivalents, are essential for accurate segregation of homologous chromosomes and requires homologous recombination and synapsis [35]. The *cisd-3*.*2(pnIs68)* mutant has a significant increase in the number of unpaired univalent chromosomes (Fig 3F and 3G). This suggests that disruption of mitochondria function can impact the proper formation of meiotic chromosomes.

Within the *C*. *elegans* germline a high level of mitochondrial DNA replication occurs indicating high number of mitochondria [36]. Mitochondrial morphology is shown to change throughout germ cell differentiation in the *C*. *elegans* germline [21]. Mutations in germline-specific mitochondrial ATPase subunits impact germline development, mitochondrial biogenesis, and disrupts the balance between mitosis and oocyte differentiation resulting in reduced fecundity [37]. Furthermore, perturbed mitochondrial bioenergetics affect germ cell differentiation and globular (immature) mitochondria become elongated [21]. The regulation of the two core signaling pathways associated with oocyte maturation (MAPK/ERK and MSP) are associated with mitochondria bioenergetics [21]. This, combined with our work, show that mitochondrial homeostasis and function are essential for progression through meiosis. It will be of interest to determine if disruption of the CISD-3 proteins lead to iron imbalance which then impacts meiosis.

In mammals including humans, the mature oocyte contains many mitochondria (estimated to be in the millions) to support the growth and development of the early embryo. Critical factors, such as mitochondrial size, function, and number as well as ROS defense processes, are associated with successful oogenesis, fertilization, and embryo development [38]. Studies suggest oxidative stress can partially cause infertility and defective spermatogenesis [39–42]. Within *C*. *elegans*, *cisd-1* dysfunction leads to hyperfused mitochondrial morphology, and higher levels of generated mitochondrial superoxide [17]. Biological cell culture studies with CISD3/MiNT knockdown revealed an increase in mitochondrial reactive species formation [5]. It will be of interest to examine the role ROS homeostasis has in *C*. *elegans* meiosis. Thus, the work conducted in *C*. *elegans* can translate to a greater understanding of the role mitochondrial homeostasis has in human fertility issues.

## Materials and methods

### Worm strains and culture conditions

Worms were raised and maintained at 20°C on Nematode Growth Media (NGM) seeded with *E*. *coli* (OP50) bacteria as a food source or HT115, *cisd-3*.*1(RNAi)* or *cisd-3*.*2(RNAi)* for RNAi experiments [16, 43, 44]. The N2 wild-type strain and *hsp-6*::*GFP* transcriptional reporter strain (SJ4100) [24] were acquired from the *C*. *elegans* Genetic Center. A CRISPR/Cas9-based approached was used to produce insertion and fluorescent reporter strains [45]. To disrupt the *cisd-3*.*2* gene and produce a transcriptional reporter, the CRISPR construct was designed so that mKATE was inserted before the *cisd*.*3*.*2* gene start site; this produced the PM58 strain [*cisd-3*.*2(pnIs64[mKATE^SEC^3xMyc]*::*cisd-3*.*2)*]III referred to as *cisd-3*.*2(pnIs64)*. The PM58 strain [*cisd-3*.*2(pnIs64[mKATE^SEC^3xMyc]*::*cisd-3*.*2)*] was collected as L1/L2 larvae, exposed to heat shock treatment (32°C for 4 hours) to remove the SEC cassette and obtain the non-roller mutant PM91 strain [*cisd-3*.*2(pnIs68 [mKATE^3xMyc]*::*cisd-3*.*2)*] (S1 Fig). The strains PM08 [*cisd-3*.*2(pnIs25*[*mKATE^SEC^3xMyc*::*cisd-3*.*2*)]III, referred to as *cisd-3*.*2(pnIs25)*::*mKATE* or CISD-3.2::mKATE and PM38 [*cisd-3*.*1(pnIs39*[mYPET^SEC^3xFlag::*cisd-3*.*1*)]III, referred to as *cisd-3*.*1(pnIs39)*::*mYPET*, or CISD-3.1::mYPET are translational fluorescent reporters. Vectors used to produce the strains include the Cas9-sgRNA vector pDD162 (Addgene plasmid #47549), FP-SEC vector pDD287 (Addgene plasmid #70685), and FP-SEC vector pDD283 (Addgene plasmid #66824). For all strains produced, the plasmid preparation, micro-injections and genome editing was verified using PCR and DNA sequencing as previously described [16]. All primers used in this study are listed in S1 Table.

### Quantitative RT-PCR

The *cisd-3*.*2* transcript within the 1-day old *cisd-3*.*2(pnIs68)* mutant and N2 wild-type adult hermaphrodite was analyzed using RT-PCR as previously described [16]. Briefly, mRNA was isolated using the following reagents; Trizol Reagent (Life Technology), NucleoSpin RNA Clean-up (Machenerey-Nagel) and TURBO DNA-free^TM^ kit (Life Technologies). cDNA was generated using Superscript III synthesis kit (Invitrogen). StepOnePlus real-time PCR system (Applied Biosystems) and PowerupSYBR Green Master Mix (Applied Biosystems) was used to carry out quantitative RT-PCR as previously described [16]. The mRNA level of the housekeeping gene Y45F10D.4 was used for normalization [46]. The relative expression levels were calculated using REST software [47]. The average of three independent biological replicates, with three technical replicates, was statistically analyzed using unpaired two-tailed t test with Welch’s correction.

### Germline development assay

The developmental progression of the germline was examined in the N2 wild-type and *cisd-3*.*2(pnIs68)* hermaphrodite using DIC Normarski microscopy. Worms were placed on a 3% agarose pad with an anesthetizing solution of .1% tricane and .01% levinmosole. Animals were analyzed using the motorized Zeiss Axioscope mot plus 2 microscope. Images were captured using the Zeiss Axiocam camera and Axio Vision 4.7.1 software. Morphological features examined include extent of germ cell proliferation, bend within the germline, and the presence of sperm and oocytes. The stage of germline development was categorized at specific time points post egg hatching (48, 65, 71 and 91 hours) (Refer to Fig 2B for categorization). Animals were categorized as Class II if the germline contained several proliferated cells beyond the Z2 and Z3 cells. Class III refers to a germline with a higher presence of proliferated cells and no bend in the germline; at this stage, the presence of vulva precursor cells was observed. Class IV refers to a germline in which the bend is observed but no oocytes are present. Class V refers to a germline in which visible oocytes are present in the diakinesis region. In three independent experiments, at least 10 animals for each genotype were examined at each time point.

### Fecundity, embryo lethality, and incidence of male assays

As previously described [16], to assess fecundity, synchronized N2 wild-type or *cisd-3*.*2(pnIs68)* animals (n = 4) were collected at the L4-to-adult molt and placed individually onto a NGM plate and allowed to lay eggs. Every 24 hours the adults were transferred to a new NGM plate and the number of progeny produced for each animal was quantified after hatching. Animals were examined until no progeny were produced. To assess embryo lethality and the high incidence of males, synchronized N2 wild-type or *cisd-3*.*2(pnIs68)* mutant hermaphrodite (N = 9) were collected at the L4-to-adult molt and transferred daily to new NGM plates until reproduction ceased. Each day, the embryo lethality was determined by quantifying the number of eggs laid and the number of unhatched eggs 24-hours post egg lay. To assess the high incidence of male (HIM) phenotype the hatched offspring were allowed to develop and the number of hermaphrodite and male progeny was quantified 48 hours after hatching.

### Lifespan measurement

Synchronized N2 wild-type or *cisd-3*.*2(pnIs68)* mutant, at the L4-to-adult hermaphrodite molt stage, were transferred to fresh NGM plates (time = 0). The N2 wild-type and *cisd-3*.*2(pnIs68)* mutant was scored daily for survivorship. The gravid hermaphrodites were transferred to a new plate every day. Non-gravid animals as they aged were transferred less frequently to minimize inducing injury. Animals were scored as dead if the worm failed to move after touching and was subsequently removed from the plate. The animals that crawled off the plate, bagged out, or showed uterine rupture were nulled. Three biological replicates were completed with a total of N = 50 animals for each experiment.

### Pharyngeal pumping and locomotion analysis

Pharyngeal and locomotion were assessed in synchronized 1-day old adult N2 wild-type or *cisd-3*.*2(pnIs68)* mutant animals (n = 10). The number of pharyngeal pumps was quantified over a 30-second period to assess pumping rate per minute. To assess locomotion, the number of body bends within one-minute intervals was quantified. A body bend is defined as a change in the direction of propagation, or the completion of one sinusoidal movement of the animal’s tail.

### Live animal microscopy analysis

To visualize worms of the specified genotypes, animals were placed in an anesthetizing solution of 0.1% tricane and 0.01% levinmosole and mounted on 3% agarose slides. Synchronized animals, at the stage indicated, were analyzed using a motorized Zeiss Axioscope mot 2 plus microscope or a Zeiss LSM 710 Confocal Scanning Microscope. To assess the impact *cisd-3*.*1(RNAi)* and/or *cisd-3*.*2(RNAi)* has on the *hsp-6*::*GFP* fluorescent reporter, synchronized animals of the F1 generation were grown to L4 larval stage on the specified RNAi food and analyzed by epifluorescence microscopy. The integrated density, area, and mean background fluorescence was computed using ImageJ. As described by others [48], the Corrected Total Worm Fluorescence (CTWF) was calculated using the following formula for Corrected Total Cell Fluorescence as described, CTWF = Integrated Density- (area of selected worm * Mean background fluorescence). Three independent experiments were conducted analyzing a total of 30 animals per RNAi treatment. Images were processed using ImageJ and Adobe Photoshop.

### Hoescht 33342 germline staining

The germline nuclei in 1-day old adult hermaphrodites were analyzed using Hoescht 33342 staining; nuclei analyzed includes those within the mitotic region, meiotic stage, oocyte region, and sperm containing spermatheca. Briefly, a synchronized population of adult hermaphrodites were fixed in ethanol for 4 minutes, rinsed with M9, and stained with Hoescht 33342 (10 μg/ml) mixed within anti-fade reagent. Animals were placed on a slide and visualized using a motorized Zeiss Axioscope microscope. Images were captured using the Zeiss Axiocam camera and Axio Vision 4.7.1 software. The mitotic region/transition zone (MT/TZ) boundary was determined by counting the number of mitotic nuclei from the distal tip to the region that contains distinct crescent-shaped nuclei morphology indicative of the leptonene/zygotene prophase I nuclei (Fig 2A) [19, 49]. Hoescht 33342 staining allows detection of nuclei within the transition zone and other meiotic stages (pachytene, diplotene, and diakinesis and the mature gametes) (Fig 2A). The oocyte nuclei, within the diakinesis region, were analyzed to quantify the number of bivalent chromosomes [50].

### Mitochondria analysis using MitoTracker

Young gravid adult animals, of the specified genotype, were incubated overnight on OP50 seeded plates with stock solution of MitoTracker freshly diluted in M9 containing 10 μM MitoTracker Red CMXRos (Invitrogen Cat #M7512) or swam for 20 minutes in 10 μM MitoTracker Green FM (Invitrogen Cat# M7514) respectively [51, 52]. After incubation with the MitoTracker molecule, animals were transferred to OP50 seeded plates to allow a 1-hour clearance of the dye from their guts. For colocalization studies, at least twenty *cisd-3*.*2(pnIs25)*::*mKATE* or *cisd-3*.*1(pnIs39)*::*mYPET* animals, were stained with the specified MitoTracker, and analyzed using Zeiss Azioscope. Images were captured using the Zeiss Axiocam camera and Axio Vision 4.7.1 software.

To analyze the mitochondria membrane potential (ΔΨM) within the oocytes of N2 wild-type and *cisd-3*.*2(pnIs68)* hermaphrodite, whole animals were stained with Mitotracker Red CMXRos as described above, and were imaged with a Zeiss LSM 710 Confocal Scanning Microscope and captured with a Zeiss LSM ZEN camera. The z-stack images (2 μm slices) of the germline were collected and combined into a single image; at least 15 animals per genotype were analyzed. Images were processed using ImageJ and Adobe Photoshop. The mean fluorescence intensity was measured for the individual secondary oocyte for the indicated genotypes [21].

### Basal oxygen consumption rate measurements

The oxygen consumption rate (OCR) was analyzed in young non-gravid hermaphrodites using the Seahorse-X24 instrument [53]. Approximately 100 N2 wild-type or *cisd-3*.*2(pnIs68)* animals were collected for each well and washed three times with M9 and allowed to settle by gravity. In the Seahorse 24-well assay plate, 500 μl/well of M9 was aliquoted; 4 blank wells and 3 wells per genotype. Experiments were repeated 10 times and oxygen consumption rates were performed using the following program: 10 cycles of (2 min mix, 2 min wait, 2 min measure). After the Seahorse program, samples were collected and the total number of animals for each well was counted to determine the OCR rate per animal.

### Statistical analysis

Animal experimental values were compared to control animals first by analyzing for Gaussian distribution using D’ Agostino & Pearson or Shapiro-Wilk normality test (alpha = 0.05, *P<*0.05). If the normality test was passed, a parametric statistical test was performed: unpaired t-test with Welch’s correction or ordinary one-way ANOVA followed by a Dunnet’s multiple comparison test. If the normality test was not passed, data was analyzed using nonparametric statistical tests; unpaired t-test or Kruskal-Wallis test followed by a Dunn’s multiple comparisons test. In the instance of the total % population, animal experimental values were compared to control animals by conducting a Fisher’s Exact Two-tailed test. For the lifespan experiment we conducted a comparison of survival curves (P<0.0001, Log-rank (Mantel-Cox) test and Gehan-Breslow-Wilcoxon test. The statistical test for each experiment is noted and the *P*-values are reported in the figure legends. All data sets are expressed as mean ± standard deviation (SD), or as percent of animals with the presence or absence of the noted phenotype of interest. Statistical analysis was conducted using Prism 8 software.

## Supporting information

S1 Fig Use of CRISPR to generate the *cisd-3*.*2(pnIs68)* mutant (strain PM91) and *cisd-2(pnIs64)* transcriptional reporter (strain PM58). Illustration of the *cisd-3*.*2* locus predicted to produce the *cisd-3*.*2* transcriptional reporter and mutant. The mKATE^SEC^3XMyc sequence was inserted upstream from the first exon disrupting *cisd-3*.*2* function. The reporter strain was exposed to heat shock to remove the SEC and gene resulting in the non-roller strain. The resulting strain produced (PM91) is likely a *cisd-3*.*2* null.
(JPG)

S2 Fig Generation of the *cisd-3*.*2* and *cisd-3*.*1* translational reporters via CRISPR. **(A)** Illustration of the *cisd-3*.*2* locus predicted to produce the *cisd-3*.*2* transcript. mKATE^SEC^3xMyc was inserted inside the last exon immediately upstream from the stop codon to produce the reporter *cisd-3*.*2(pnIs25[mKATE^SEC^3xMyc*::*cisd-3*.*2])* strain (PM08). **(B)** Illustration of the *cisd-3*.*1* locus predicted to produce the *cisd-3*.*1* transcript. mYPET ^SEC^3xFlag was inserted inside the last exon immediately upstream from the stop codon to produce the reporter *cisd-3*.*1(pnIs39[mYPET^SEC^3xFlag*::*cisd-3*.*1])* reporter strain (PM38).
(TIF)

S3 Fig The *cisd-3*.*2(pnIs68)* mutant has a decrease in lifespan. The lifespan of *cisd-3*.*2(pnIs68)* (median 8 days), relative to N2 wild-type worms (median 19 days), (Comparison of survival curves (P<0.0001, Log-rank (Mantel-Cox) test, Gehan-Breslow-Wilcoxon test).
(TIF)

S1 Table Primers used in this study. (DOCX)

S2 Table Data used to produce graphs and run statistical analysis. (XLSX)

## References

[1] Nechushtai R, Conlan AR, Harir Y, Song L, Yogev O, Eisenberg-Domovich Y, et al Characterization of Arabidopsis NEET reveals an ancient role for NEET proteins in iron metabolism. Plant Cell. 2012;24(5):2139–54. 10.1105/tpc.112.097634 22562611 PMC3442592

[2] Paddock ML, Wiley SE, Axelrod HL, Cohen AE, Roy M, Abresch EC, et al MitoNEET is a uniquely folded 2Fe 2S outer mitochondrial membrane protein stabilized by pioglitazone. Proc Natl Acad Sci U S A. 2007;104(36):14342–7. 10.1073/pnas.0707189104 17766440 PMC1963346

[3] Sohn YS, Tamir S, Song L, Michaeli D, Matouk I, Conlan AR, et al NAF-1 and mitoNEET are central to human breast cancer proliferation by maintaining mitochondrial homeostasis and promoting tumor growth. Proc Natl Acad Sci U S A. 2013;110(36):14676–81. 10.1073/pnas.1313198110 23959881 PMC3767537

[4] Wiley SE, Murphy AN, Ross SA, van der Geer P, Dixon JE. MitoNEET is an iron-containing outer mitochondrial membrane protein that regulates oxidative capacity. Proc Natl Acad Sci U S A. 2007;104(13):5318–23. 10.1073/pnas.0701078104 17376863 PMC1838440

[5] Lipper CH, Karmi O, Sohn YS, Darash-Yahana M, Lammert H, Song L, et al Structure of the human monomeric NEET protein MiNT and its role in regulating iron and reactive oxygen species in cancer cells. Proc Natl Acad Sci U S A. 2018;115(2):272–7. 10.1073/pnas.1715842115 29259115 PMC5777063

[6] Chang NC, Nguyen M, Germain M, Shore GC. Antagonism of Beclin 1-dependent autophagy by BCL-2 at the endoplasmic reticulum requires NAF-1. EMBO J. 2010;29(3):606–18. 10.1038/emboj.2009.369 20010695 PMC2830692

[7] Mittler R, Darash-Yahana M, Sohn YS, Bai F, Song L, Cabantchik IZ, et al NEET Proteins: A New Link Between Iron Metabolism, Reactive Oxygen Species, and Cancer. Antioxid Redox Signal. 2019;30(8):1083–95. 10.1089/ars.2018.7502 . 29463105 PMC10625470

[8] Rondinelli M, Novara F, Calcaterra V, Zuffardi O, Genovese S. Wolfram syndrome 2: a novel CISD2 mutation identified in Italian siblings. Acta Diabetol. 2015;52(1):175–8. 10.1007/s00592-014-0648-1 . 25371195

[9] Rouzier C, Moore D, Delorme C, Lacas-Gervais S, Ait-El-Mkadem S, Fragaki K, et al A novel CISD2 mutation associated with a classical Wolfram syndrome phenotype alters Ca2+ homeostasis and ER-mitochondria interactions. Hum Mol Genet. 2017;26(9):1786 10.1093/hmg/ddx130 28475771 PMC5411737

[10] al-Sheyyab M, Jarrah N, Younis E, Shennak MM, Hadidi A, Awidi A, et al Bleeding tendency in Wolfram syndrome: a newly identified feature with phenotype genotype correlation. Eur J Pediatr. 2001;160(4):243–6. 10.1007/s004310000704 . 11317648

[11] Mozzillo E, Delvecchio M, Carella M, Grandone E, Palumbo P, Salina A, et al A novel CISD2 intragenic deletion, optic neuropathy and platelet aggregation defect in Wolfram syndrome type 2. BMC Med Genet. 2014;15:88 10.1186/1471-2350-15-88 25056293 PMC4121299

[12] Inupakutika MA, Sengupta S, Nechushtai R, Jennings PA, Onuchic JN, Azad RK, et al Phylogenetic analysis of eukaryotic NEET proteins uncovers a link between a key gene duplication event and the evolution of vertebrates. Sci Rep. 2017;7:42571 10.1038/srep42571 28205535 PMC5311916

[13] Sengupta S, Nechushtai R, Jennings PA, Onuchic JN, Padilla PA, Azad RK, et al Phylogenetic analysis of the CDGSH iron-sulfur binding domain reveals its ancient origin. Sci Rep. 2018;8(1):4840 10.1038/s41598-018-23305-6 29556009 PMC5859297

[14] Ellis HM, Horvitz HR. Genetic control of programmed cell death in the nematode C. elegans. Cell. 1986;44(6):817–29. Epub 1986/03/28. 10.1016/0092-8674(86)90004-8 . 3955651

[15] Gumienny TL, Lambie E, Hartwieg E, Horvitz HR, Hengartner MO. Genetic control of programmed cell death in the Caenorhabditis elegans hermaphrodite germline. Development. 1999;126(5):1011–22. . 9927601 10.1242/dev.126.5.1011

[16] King SD, Gray CF, Song L, Nechushtai R, Gumienny TL, Mittler R, et al The cisd gene family regulates physiological germline apoptosis through ced-13 and the canonical cell death pathway in Caenorhabditis elegans. Cell Death Differ. 2019;26(1):162–78. 10.1038/s41418-018-0108-5 29666474 PMC6294797

[17] Hsiung KC, Liu KY, Tsai TF, Yoshina S, Mitani S, Chin-Ming Tan B, et al Defects in CISD-1, a mitochondrial iron-sulfur protein, lower glucose level and ATP production in Caenorhabditis elegans. Biomed J. 2020;43(1):32–43. 10.1016/j.bj.2019.07.009 32200954 PMC7090286

[18] Hubbard EJ, Greenstein D. Introduction to the germ line. WormBook. 2005:1–4. 10.1895/wormbook.1.18.1 18050415 PMC4781435

[19] Kimble J, Crittenden SL. Germline proliferation and its control. WormBook. 2005:1–14. 10.1895/wormbook.1.13.1 18050413 PMC4781503

[20] Crittenden SL, Kimble J. Analysis of the C. elegans germline stem cell region. Methods Mol Biol. 2008;450:27–44. 10.1007/978-1-60327-214-8_2 . 18370049

[21] Charmpilas N, Tavernarakis N. Mitochondrial maturation drives germline stem cell differentiation in Caenorhabditis elegans. Cell Death Differ. 2020;27(2):601–17. 10.1038/s41418-019-0375-9 . 31217501 PMC7206027

[22] Pitt JN, Schisa JA, Priess JR. P granules in the germ cells of Caenorhabditis elegans adults are associated with clusters of nuclear pores and contain RNA. Dev Biol. 2000;219(2):315–33. 10.1006/dbio.2000.9607 . 10694425

[23] Labrousse AM, Zappaterra MD, Rube DA, van der Bliek AM. C. elegans dynamin-related protein DRP-1 controls severing of the mitochondrial outer membrane. Mol Cell. 1999;4(5):815–26. 10.1016/s1097-2765(00)80391-3 . 10619028

[24] Yoneda T, Benedetti C, Urano F, Clark SG, Harding HP, Ron D. Compartment-specific perturbation of protein handling activates genes encoding mitochondrial chaperones. J Cell Sci. 2004;117(Pt 18):4055–66. 10.1242/jcs.01275 . 15280428

[25] Sheftel AD, Stehling O, Pierik AJ, Elsasser HP, Muhlenhoff U, Webert H, et al Humans possess two mitochondrial ferredoxins, Fdx1 and Fdx2, with distinct roles in steroidogenesis, heme, and Fe/S cluster biosynthesis. Proc Natl Acad Sci U S A. 2010;107(26):11775–80. 10.1073/pnas.1004250107 20547883 PMC2900682

[26] Miller WL. Minireview: regulation of steroidogenesis by electron transfer. Endocrinology. 2005;146(6):2544–50. 10.1210/en.2005-0096 . 15774560

[27] Shi Y, Ghosh M, Kovtunovych G, Crooks DR, Rouault TA. Both human ferredoxins 1 and 2 and ferredoxin reductase are important for iron-sulfur cluster biogenesis. Biochim Biophys Acta. 2012;1823(2):484–92. 10.1016/j.bbamcr.2011.11.002 22101253 PMC3546607

[28] Durieux J, Wolff S, Dillin A. The cell-non-autonomous nature of electron transport chain-mediated longevity. Cell. 2011;144(1):79–91. 10.1016/j.cell.2010.12.016 21215371 PMC3062502

[29] Haynes CM, Fiorese CJ, Lin YF. Evaluating and responding to mitochondrial dysfunction: the mitochondrial unfolded-protein response and beyond. Trends Cell Biol. 2013;23(7):311–8. 10.1016/j.tcb.2013.02.002 23489877 PMC3700555

[30] Liu Y, Samuel BS, Breen PC, Ruvkun G. Caenorhabditis elegans pathways that surveil and defend mitochondria. Nature. 2014;508(7496):406–10. 10.1038/nature13204 24695221 PMC4102179

[31] Nargund AM, Pellegrino MW, Fiorese CJ, Baker BM, Haynes CM. Mitochondrial import efficiency of ATFS-1 regulates mitochondrial UPR activation. Science. 2012;337(6094):587–90. 10.1126/science.1223560 22700657 PMC3518298

[32] Fox PM, Schedl T. Analysis of Germline Stem Cell Differentiation Following Loss of GLP-1 Notch Activity in Caenorhabditis elegans. Genetics. 2015;201(1):167–84. 10.1534/genetics.115.178061 26158953 PMC4566261

[33] Albertson DG, Rose AM, Villeneuve AM. Chromosome Organization, Mitosis and Meiosis. Riddle DL, Blumenthal T, Meyer BJ, Priess JR, editors: Cold Spring Harbor Mongraph Series; 1997. 21413226

[34] Villeneuve AM. A cis-acting locus that promotes crossing over between X chromosomes in Caenorhabditis elegans. Genetics. 1994;136(3):887–902. 8005443 10.1093/genetics/136.3.887 PMC1205894

[35] Tsai JH, McKee BD. Homologous pairing and the role of pairing centers in meiosis. J Cell Sci. 2011;124(Pt 12):1955–63. 10.1242/jcs.006387 . 21625006

[36] Bratic I, Hench J, Henriksson J, Antebi A, Burglin TR, Trifunovic A. Mitochondrial DNA level, but not active replicase, is essential for Caenorhabditis elegans development. Nucleic Acids Res. 2009;37(6):1817–28. 10.1093/nar/gkp018 19181702 PMC2665216

[37] Kawasaki I, Hanazawa M, Gengyo-Ando K, Mitani S, Maruyama I, Iino Y. ASB-1, a germline-specific isoform of mitochondrial ATP synthase b subunit, is required to maintain the rate of germline development in Caenorhabditis elegans. Mech Dev. 2007;124(3):237–51. 10.1016/j.mod.2006.11.004 . 17223323

[38] Chappel S. The role of mitochondria from mature oocyte to viable blastocyst. Obstet Gynecol Int. 2013;2013:183024 10.1155/2013/183024 23766762 PMC3671549

[39] Bedaiwy MA, Goldberg JM, Falcone T, Singh M, Nelson D, Azab H, et al Relationship between oxidative stress and embryotoxicity of hydrosalpingeal fluid. Hum Reprod. 2002;17(3):601–4. 10.1093/humrep/17.3.601 . 11870110

[40] Sharma RK, Agarwal A. Role of reactive oxygen species in gynecologic diseases. Reprod Med Biol. 2004;3(4):177–99. 10.1111/j.1447-0578.2004.00068.x 29699197 PMC5904640

[41] Agarwal A, Saleh RA, Bedaiwy MA. Role of reactive oxygen species in the pathophysiology of human reproduction. Fertil Steril. 2003;79(4):829–43. 10.1016/s0015-0282(02)04948-8 . 12749418

[42] Aitken RJ, De Iuliis GN, Finnie JM, Hedges A, McLachlan RI. Analysis of the relationships between oxidative stress, DNA damage and sperm vitality in a patient population: development of diagnostic criteria. Hum Reprod. 2010;25(10):2415–26. 10.1093/humrep/deq214 . 20716559

[43] Brenner S. The genetics of Caenorhabditis elegans. Genetics. 1974;77(1):71–94. Epub 1974/05/01. 4366476 10.1093/genetics/77.1.71 PMC1213120

[44] Timmons L, Court DL, Fire A. Ingestion of bacterially expressed dsRNAs can produce specific and potent genetic interference in Caenorhabditis elegans. Gene. 2001;263(1–2):103–12. 10.1016/s0378-1119(00)00579-5 . 11223248

[45] Dickinson DJ, Pani AM, Heppert JK, Higgins CD, Goldstein B. Streamlined Genome Engineering with a Self-Excising Drug Selection Cassette. Genetics. 2015;200(4):1035–49. 10.1534/genetics.115.178335 26044593 PMC4574250

[46] Hoogewijs D, Houthoofd K, Matthijssens F, Vandesompele J, Vanfleteren JR. Selection and validation of a set of reliable reference genes for quantitative sod gene expression analysis in C. elegans. BMC Mol Biol. 2008;9:9 10.1186/1471-2199-9-9 18211699 PMC2254638

[47] Pfaffl MW, Horgan GW, Dempfle L. Relative expression software tool (REST) for group-wise comparison and statistical analysis of relative expression results in real-time PCR. Nucleic Acids Res. 2002;30(9):e36 10.1093/nar/30.9.e36 11972351 PMC113859

[48] McCloy RA, Rogers S, Caldon CE, Lorca T, Castro A, Burgess A. Partial inhibition of Cdk1 in G 2 phase overrides the SAC and decouples mitotic events. Cell Cycle. 2014;13(9):1400–12. 10.4161/cc.28401 24626186 PMC4050138

[49] Crittenden SL, Leonhard KA, Byrd DT, Kimble J. Cellular analyses of the mitotic region in the Caenorhabditis elegans adult germ line. Mol Biol Cell. 2006;17(7):3051–61. 10.1091/mbc.e06-03-0170 16672375 PMC1552046

[50] Dernburg AF, McDonald K, Moulder G, Barstead R, Dresser M, Villeneuve AM. Meiotic recombination in C. elegans initiates by a conserved mechanism and is dispensable for homologous chromosome synapsis. Cell. 1998;94(3):387–98. 10.1016/s0092-8674(00)81481-6 . 9708740

[51] Gitschlag BL, Kirby CS, Samuels DC, Gangula RD, Mallal SA, Patel MR. Homeostatic Responses Regulate Selfish Mitochondrial Genome Dynamics in C. elegans. Cell Metab. 2016;24(1):91–103. 10.1016/j.cmet.2016.06.008 27411011 PMC5287496

[52] Kassahun H, SenGupta T, Schiavi A, Maglioni S, Skjeldam HK, Arczewska K, et al Constitutive MAP-kinase activation suppresses germline apoptosis in NTH-1 DNA glycosylase deficient C. elegans. DNA Repair (Amst). 2018;61:46–55. 10.1016/j.dnarep.2017.11.009 . 29202295

[53] Dancy BM, Kayser EB, Sedensky MM, Morgan PG. Live worm respiration in 24-well format. Worm Breeder’s Gazzette. 2013;19(4).

