# Supplementary figures and images for: The mitochondrial localized CISD-3.1/CISD-3.2 proteins are required to maintain normal germline structure and function in Caenorhabditis elegans
Source: PLoS One. 2021 Feb 5;16(2):e0245174. doi: 10.1371/journal.pone.0245174 (PMC7864470; doi:10.1371/journal.pone.0245174)

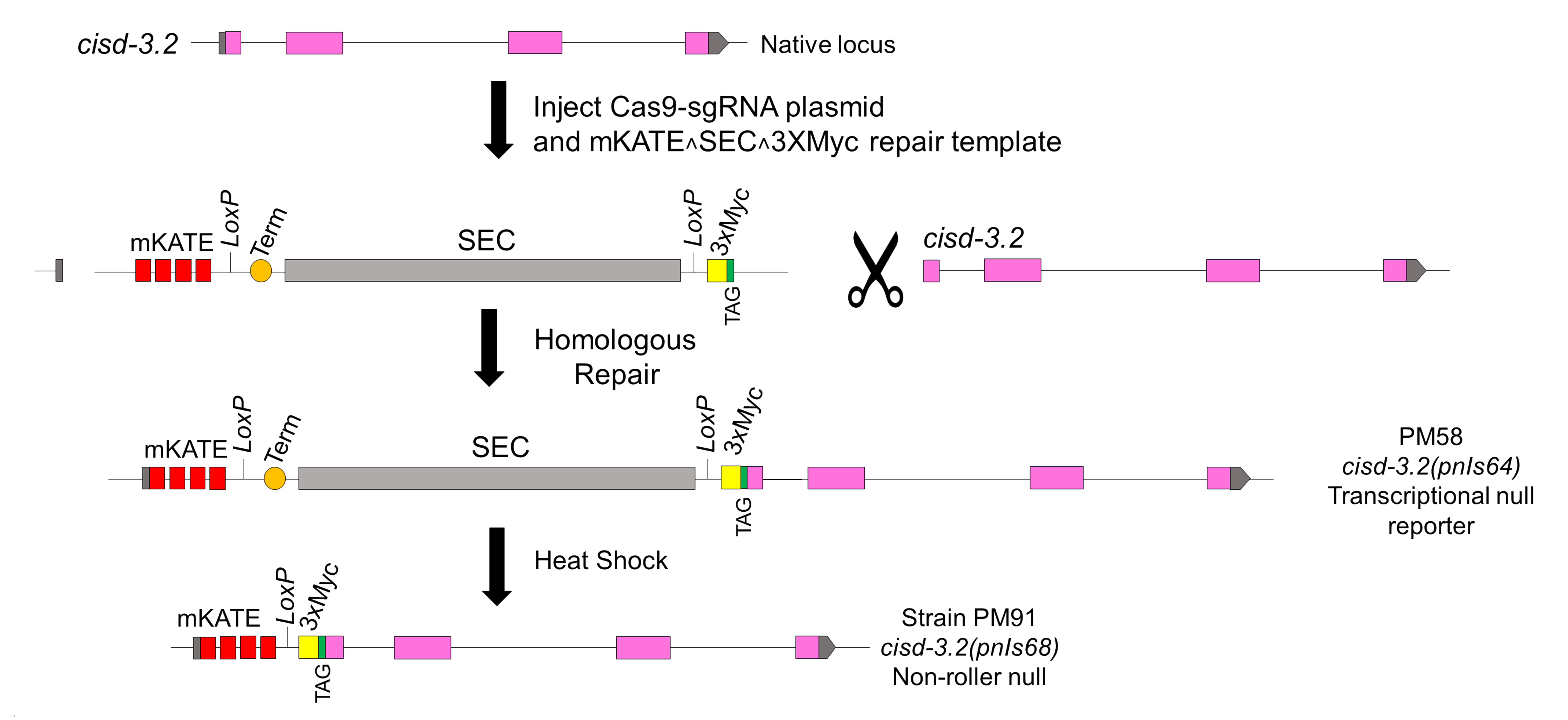

Supplement: S1 Fig — Illustration of the cisd-3.2 locus predicted to produce the cisd-3.2 transcriptional reporter and mutant. The mKATE^SEC^3XMyc sequence was inserted upstream from the first exon disrupting cisd-3.2 function. The reporter strain was exposed to heat shock to remove the SEC and gene resulting in the non-roller strain. The resulting strain produced (PM91) is likely a cisd-3.2 null. (JPG) [file pone.0245174.s001.jpg]

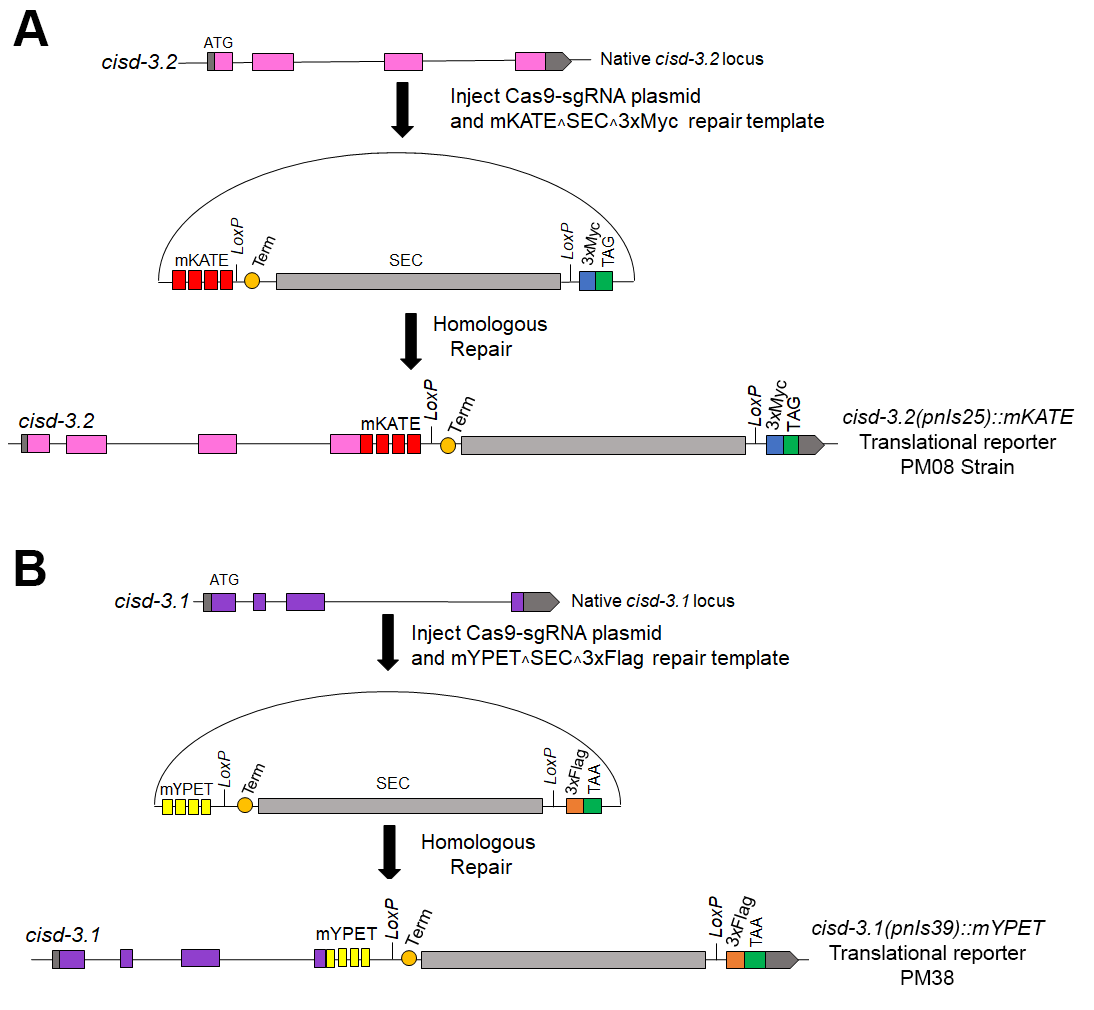

Supplement: S2 Fig — (A) Illustration of the cisd-3.2 locus predicted to produce the cisd-3.2 transcript. mKATE^SEC^3xMyc was inserted inside the last exon immediately upstream from the stop codon to produce the reporter cisd-3.2(pnIs25[mKATE^SEC^3xMyc::cisd-3.2]) strain (PM08). (B) Illustration of the cisd-3.1 locus predicted to produce the cisd-3.1 transcript. mYPET ^SEC^3xFlag was inserted inside the last exon immediately upstream from the stop codon to produce the reporter cisd-3.1(pnIs39[mYPET^SEC^3xFlag::cisd-3.1]) reporter strain (PM38). (TIF) [file pone.0245174.s002.tif]

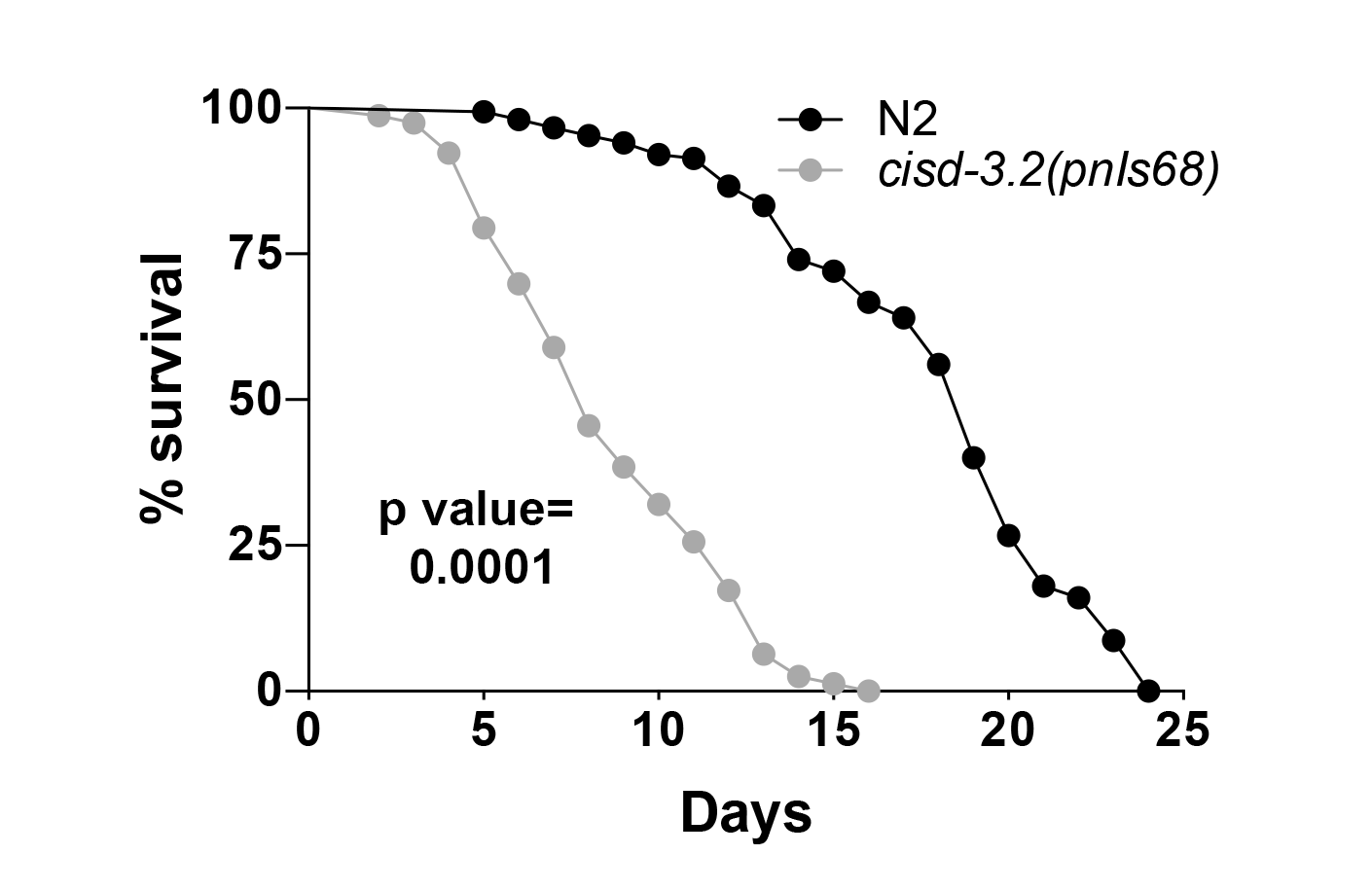

Supplement: S3 Fig — The lifespan of cisd-3.2(pnIs68) (median 8 days), relative to N2 wild-type worms (median 19 days), (Comparison of survival curves (P<0.0001, Log-rank (Mantel-Cox) test, Gehan-Breslow-Wilcoxon test). (TIF) [file pone.0245174.s003.tif]
