# Supplementary material for: The mitochondrial localized CISD-3.1/CISD-3.2 proteins are required to maintain normal germline structure and function in Caenorhabditis elegans
Source: PLoS One. 2021 Feb 5;16(2):e0245174. doi: 10.1371/journal.pone.0245174 (PMC7864470; doi:10.1371/journal.pone.0245174)
Supplement: S1 Table — (DOCX) [file pone.0245174.s004.docx]

| **S1 Table. Primers used in this study** | | | |
| --- | --- | --- | --- |
| **Gene (allele)** | **Primer Use** | **Forward Sequence** | **Reverse Sequence** |
| *cisd-3.2(pnIs64)* | Cas-9-sgRNA target N-term knock-in | aggtcgaattgagccatgctgttttagagctagaaatagcaagt | caagacatctcgcaatagg |
| *cisd-3.2(pnIs64)* | Left HA | acgttgtaaaacgacggccagtcgccggcaactaggagtgtcgtagtttttgg | catgttttctttaatgagctcggagaccatggctcaattcgacctgaaaatcg |
| *cisd-3.2(pnIs64)* | Right HA | gagcagaagttgatcagcgaggaagacttgtagatgctcgaaagaacgtgccaaaagc | tcacacaggaaacagctatgaccatgttattcgcaaaaaatctttatttccagcg |
| *cisd-3.2(pnIs64)* | Verification of N term knock-in left side | aaatcggcaaaaaagtgtaaaa | tcggaggtgcacttgaagt |
| *cisd-3.2(pnIs64)* | Verification of N term knock-in right side | gtgctcccaccccctatttt | ggccgccaatctattttgagcg |
| *cisd-3.2(pnIs68)* | qRT-PCR | acgctgatgtatccaaagc | caactcctgcgtaaactgg |
| *cisd-3.2(pnIs68)* | Verification of SEC cassette removal | aaatcggcaaaaaagtgtaaaa | ggccgccaatctattttgagcg |
| *cisd-3.2(pnIs25)* | Cas-9-sgRNA target C-term knock-in | cagcgtagtctccggactacgttttagagctagaaatagcaagt | caagacatctcgcaatagg |
| *cisd-3.2(pnIs25)* | LHA | gtcacgacgttgtaaaacgacggccagtcgccaagaaaatcattaaattcatg | catcgatgctcctgaggctcccgatgctccctggaatccaccatttttcggct |
| *cisd-3.2(pnIs25)* | RHA | gagcagaagttgatcagcgaggaagacttgtagtccggagactacgctgagacagcagccga | ggaaacagctatgaccatgttatcgatttctattccatttttcgcatatttttaa |
| *cisd-3.2(pnIs25)* | Verification of C term knock-in left side | cgatttttcactcacatttgctca | tcggaggtgcacttgaagt |
| *cisd-3.2(pnIs25)* | Verification of C term knock-in right side | gggagccggatctgaacaaaaa | ctagggggtactgtagctccg |
| *cisd-3.1(pnIs39)* | Cas-9-sgRNA target C-term knock-in | gacctaaaactatatggagtgttttagagctagaaatagcaagt | caagacatctcgcaatagg |
| *cisd-3.1(pnIs39)* | LHA | acgttgtaaaacgacggccagtcgccggcatttttttaaattaacgtaaaaaa | catcgatgctcctgaggctcccgatgctccatcgaaaagtccagctttcttgt |
| *cisd-3.1(pnIs39)* | RHA | cgtgattacaaggatgacgatgacaagagataattatgaacaactccatatagttttagg | ggaaacagctatgaccatgttatcgatttctccgtgtcttgagaagtttggcaag |
| *cisd-3.1(pnIs39)* | Verification of C term knock-in left side | ccgtcctcgtttcaatggaatcac | ctccctctccggagacgg |
| *cisd-3.1(pnIs39)* | Verification of C term knock-in right side | gcgtgactacaaggacgacga | gcataacccttcgcgtgtcc |
| *cisd-3.1-F1* | Primer for DNA sequencing | gcaatgaggcgcagtgca |  |
| *cisd-3.1-F2* | Primer for DNA sequencing | agctaccaactaaggtattca |  |
| *cisd-3.1-F3* | Primer for DNA sequencing | gtacaattccagagagttgt |  |
| *cisd-3.1-F4* | Primer for DNA sequencing | catccagttgttccatccg |  |
| *cisd-3.1-F5* | Primer for DNA sequencing | cattcgaattgaaatcagtcatc |  |
| *cisd-1* | Primer for DNA sequencing | gtccacgaggctgaagatt | gtgaatctgatgactgcaaac |
| *cisd-3.1* | qRT-PCR | ggaatacggtctgcaagga | catgtgaaccttagttggtagc |
| *cisd-1* | qRT-PCR | gctcttattggataccttgttgg | cttattcgagtcgagtgga |
| Y45F10D.4 | qRT-PCR | gtcgcttcaaatcagttcagc | gttcttgtcaagtgatccgaca |
